# Acute phase inflammation is characterized by rapid changes in plasma/peritoneal fluid *N*-glycosylation in mice

**DOI:** 10.1007/s10719-015-9648-9

**Published:** 2016-02-29

**Authors:** Yoann Rombouts, Hulda S. Jónasdóttir, Agnes L. Hipgrave Ederveen, Karli R. Reiding, Bas C. Jansen, Jona Freysdottir, Ingibjörg Hardardottir, Andreea Ioan-Facsinay, Martin Giera, Manfred Wuhrer

**Affiliations:** Center for Proteomics and Metabolomics, Leiden University Medical Center, Leiden, The Netherlands; Department of Rheumatology, Leiden University Medical Center, Leiden, The Netherlands; Institut de Pharmacologie et de Biologie Structurale, Université de Toulouse, CNRS, UPS, Toulouse, France; Faculty of Medicine, Biomedical Center, School of Health Sciences, University of Iceland, Reykjavik, Iceland; Department of Immunology and Center for Rheumatology Research, Landspitali-The National University Hospital of Iceland, Reykjavik, Iceland; Division of BioAnalytical Chemistry, VU University Amsterdam, Amsterdam, The Netherlands

**Keywords:** Zymosan-induced peritonitis, Mouse, *N*-glycosylation changes, Plasma, Peritoneal fluid

## Abstract

Murine zymosan-induced peritonitis is a widely used model for studying the molecular and cellular events responsible for the initiation, persistence and/or resolution of inflammation. Among these events, it is becoming increasingly evident that changes in glycosylation of proteins, especially in the plasma and at the site of inflammation, play an important role in the inflammatory response. Using matrix-assisted laser desorption/ionization time-of-flight mass spectrometry (MALDI-TOF-MS)-based glycosylation profiling, we investigated the qualitative and quantitative effect of zymosan-induced peritonitis on *N*-glycosylation in mouse plasma and peritoneal fluid. Our results show that both *N*-glycomes exhibit highly similar glycosylation patterns, consisting mainly of diantennary and triantennary complex type *N*-glycans with high levels (>95 %) of galactosylation and sialylation (mostly NeuGc) and a medium degree of core fucosylation (30 %). Moreover, MS/MS structural analysis, assisted by linkage-specific derivatization of sialic acids, revealed the presence of *O*-acetylated sialic acids as well as disialylated antennae (“branching sialylation”) characterized by the presence of α2-6-linked NeuGc on the GlcNAc of the NeuGcα2-3-Galβ1-3-GlcNAc terminal motif. A significant decrease of (core) fucosylation together with an increase of both α2-3-linked NeuGc and “branching sialylation” were observed in *N*-glycomes of mice challenged with zymosan, but not in control mice injected with PBS. Importantly, substantial changes in glycosylation were already observed 12 h after induction of peritonitis, thereby demonstrating an unexpected velocity of the biological mechanisms involved.

**Electronic supplementary material:**

The online version of this article (doi:10.1007/s10719-015-9648-9) contains supplementary material, which is available to authorized users.

## Introduction

Acute inflammation is a fundamental immune response that occurs as a reaction to microbial infection or tissue injury, and involves a complex sequence of events on both cellular and molecular levels. It can be roughly divided into two phases, the initial pro-inflammatory and the latter pro-resolving phase. The pro-inflammatory phase of the response is initiated by cells already present in the tissue, particularly resident macrophages and mast cells [1, 2]. Pro-inflammatory mediators, such as eicosanoids and cytokines, are released upon activation of these cells, driving the recruitment and activation of granulocytes [3, 4]. The extent of inflammation, the specific type of leukocytes, and the produced mediators are tissue specific as well as dependent on the nature of the inflammatory stimulus [5, 6]. For the initial pro-inflammatory phase cytokines (e.g. IL-1, IL-6), acute phase proteins (APP) (e.g. C-reactive protein) and eicosanoids (e.g. prostaglandins, leukotrienes) have been proven relevant [4, 7, 8]. As for the pro-inflammatory phase, specific cells, proteins and molecules have been described for the pro-resolution phase. These include resolution-promoting macrophages, proteins of the lipoxygenase family, anti-inflammatory cytokines such as IL-4 and IL-10, as well as lipid mediators like lipoxins and E- and D-series resolvins [9]. Both the inflammatory and resolution phases are tightly controlled/connected and ideally lead to tissue homeostasis. However, uncontrolled inflammatory responses can cause local damage/scarring possibly leading to chronic injury [10, 11]. Such uncontrolled, non-resolving inflammation is associated with many diseases, including cancer, cardiovascular and the classic inflammatory diseases, such as rheumatoid arthritis and inflammatory bowel disease [12]. The resolution of acute inflammation and the return to homeostasis are thus essential.

In addition to the aforementioned mechanisms, glycosylation of proteins influences many immunological processes. This can be, among other means, by regulating half-life and modulating the interaction of glycoproteins with carbohydrate recognizing receptors (e.g. selectins) as well as by inducing conformation changes of immunomodulatory proteins [13–15]. As such, it is no surprise that inflammation has shown to be closely associated with changes in glycosylation (galactosylation, fucosylation, sialylation and bisection) of major plasma proteins including immunoglobulin G (IgG) and APP (e.g. alpha-1 antitrypsin) [13, 16, 17]. The abundance of these proteins leads to changes in glycosylation being detectable in the total plasma *N*-glycome (TPNG) [18, 19], even though these changes are in part obscured by other protein glycosylation. For example, significant changes in both IgG glycosylation and TPNG were observed in patients only a day after cardiac surgery [18]. This makes the analysis of complex biofluids an interesting direction for fundamental research and biomarker discovery, particularly with respect to inflammatory processes. We have recently developed a robust analytical method allowing the high-throughput profiling of the human plasma *N*-glycome by MALDI-TOF-MS [20, 21]. This approach relies on the linkage-specific chemical derivatization of sialic acids enabling the discrimination of α2-6-linked and α2-3/α2-8-linked sialic acids. Of note, far from being restricted to the analysis of human plasma *N*-glycome, this method is also suitable for glycome profiling of other fluids and glycoprotein samples and for instance was recently applied for studying glycosylation changes on IgG during pregnancy [22].

The most frequently used model for investigating self-resolving acute inflammation is the zymosan-induced peritonitis (ZIP) model, in which an insoluble polysaccharide prepared from the cell wall of *Saccharomyces cerevisiae* is injected into the peritoneum of mice [23–25]. Several investigators have studied leukocyte recruitment, pro- and anti-inflammatory lipid mediators, chemokines, cytokines as well as metabolic profiles during inflammation using this model [25–27]. However, to our knowledge no study has yet addressed a comprehensive analysis of glycomic changes during the acute self-resolving inflammation induced by zymosan.

In the present study, we used the high-throughput MS-based method described above for the comprehensive profiling of the *N*-glycosylation in plasma and peritoneal fluid of mice during the course of acute inflammation induced by zymosan challenge.

## Materials and methods

### Zymosan-induced peritonitis and immunological profiles

Female C57BL/6 mice weighing 18–20 g (Taconic Europe, Ejby, Denmark) were injected intraperitoneally (*i.p.*) with 0.5 mL of 2 mg/mL zymosan A (Sigma Aldrich, Steinheim, Germany) solubilized in low-endotoxin sterile PBS (1 mg/mouse) or with vehicle (control). At time points 0, 2, 4, 12, 24, 48, and 72 h after the injection of zymosan the mice were anesthetized with isoflurane and the peritoneal cavity lavaged with 5 mL ice-cold PBS without calcium and magnesium. Following cervical dislocation blood was collected into EDTA tubes. The blood was spun at 2000×g for 10 min, the plasma aliquoted and stored under nitrogen at −80 °C until analysis. The peritoneal lavage was spun at 225×g for 10 min, the fluid was collected, aliquoted and stored under nitrogen at −80 °C until analysis. The protein concentration was determined using the Bradford method for both plasma and peritoneal fluid. The peritoneal cells were resuspended in 1 mL PBS and counted using a Countess automated cell counter. An aliquot was kept for FACS analysis and the remaining cells stored under nitrogen at −80 °C until analysis. The study was approved by the Experimental Animal Committee, Ministry for the Environment in Iceland. The zymosan-induced peritonitis model was carried out twice (*n* = *6*) and the control experiment once (*n* = *3*).

### FACS analysis

For phenotypic characterization, 3 × 10^5^ peritoneal cells were stained with fluorochrome-labeled monoclonal antibodies against Ly6G, CD11b (BD Bioscience, San Jose, CA), CXCR2 and F4/80 (BioLegend, San Diego, CA). Ten thousand cells were collected using a Navios flow cytometer (Beckman Coulter, Bromma, Sweden) and the Kaluza program (Beckman Coulter) was used for analysis. Neutrophils were identified as CXCR2^+^Ly6G^+^ cells and macrophages as F4/80^+^CD11b^+^ cells.

### Measurement of interleukin-6 and leukotriene B_4_ concentrations in the peritoneal fluid of mice

Peritoneal fluid interleukin 6 (IL-6) concentration was determined using ELISA technology according to the manufacturer’s instructions (Mouse IL-6 ELISA Kit, BD Biosciences, Breda, The Netherlands, # 555,240). Leukotriene B_4_ (LTB_4_) was analyzed by solid phase extraction followed by LC-MS/MS analysis according to published protocols [28, 29]. One mL peritoneal lavage was worked up, avoiding the *n*-hexane wash step, dissolving the sample in 200 μL 40 % MeOH for LC-MS/MS analysis. Both IL-6 and LTB_4_ concentrations were determined for one of the two zymosan-induced peritonitis experiments.

### *N*-Glycan release from mouse plasma and peritoneal fluid

*N*-Glycans were released from the protein fraction as previously described [30]. To this end, 5 μL of plasma were denatured with 10 μL 2 % sodium dodecyl sulfate (SDS; Merck, Darmstadt, Germany) and incubated for 10 min at 60 °C. The subsequent release step was performed by adding 10 μL of a mixture containing 2 % Nonidet P-40 substitute (NP-40; Sigma-Aldrich) and 0.5 mU recombinant peptide-*N*-glycosidase F (PNGase F; Roche Diagnostics, Mannheim, Germany) in 2.5× PBS, followed by overnight incubation at 37 °C. In case of peritoneal fluid, 100 μL of sample were dried by vacuum centrifugation and solubilized in 10 μL Milli-Q (MQ) water followed by 10 min sonication. The denaturing step was performed by adding 20 μL 2 % SDS for 10 min at 60 °C. The *N*-glycans were released by adding 20 μL of a mixture containing 2 % NP-40 and 1 mU PNGase F in 2.5× PBS, followed by overnight incubation at 37 °C.

### *N*-Glycan derivatization and purification

The released *N*-glycans were derivatized by ethyl esterification as described [20]. This derivatization method allows a selective ethyl esterification of α2-6-linked sialic acids and lactonization of α2-3-linked sialic acids. The derivatization reagent was prepared by mixing 1-ethyl-3-(3-dimethylaminopropyl) carbodiimide (EDC; Fluorochem, Hadfield, UK) with 1-hydroxybenzotriazole (HOBt; Sigma-Aldrich), resulting in a final concentration of 0.25 M in ethanol (Merck) for both reagents. For each sample, 2 μL of released *N*-glycans were added to 20 μL of derivatization reagent in a 96-well plate. The plate was sealed to prevent evaporation and incubated for 1 h at 37 °C. Following cool down to room temperature, the derivatized glycans were purified by hydrophilic interaction chromatography (HILIC) using pipette tips filled with cotton as stationary phase [31]. Briefly, 20 μL of acetonitrile (ACN; Biosolve, Valkenswaard, The Netherlands) were added to the reaction mixture. The tips (cotton inserted) were washed three times with 20 μL of MQ water and three times with 20 μL of 85 % ACN followed by sample loading by pipetting twenty times up and down. Finally, cotton tips were washed by pipetting three times with 20 μL 85 % ACN containing 1 % trifluoroacetic acid (TFA; Merck) and three times with 20 μL 85 % ACN, followed by elution of samples in 10 μL MQ.

### Matrix-assisted laser desorption/ionization time-of-flight mass spectrometry (MALDI-TOF-MS) profiling of total plasma *N*-glycome and total peritoneal fluid *N*-glycome

Each sample was measured once by mixing 5 μL of purified derivatized glycans with 0.5 μL of 5 mg/mL 2,5-dihydroxybenzoic acid (Bruker Daltonics, Bremen, Germany) 1 mM NaOH in 50 % ACN on a MTP AnchorChip 800/384 TF MALDI target (Bruker Daltonics) and left to dry at room temperature. Subsequently, the dried sample spots were recrystallized by adding 0.2 μL ethanol. All analyses were performed on an UltraFlextreme MALDI-TOF/TOF-MS equipped with a Smartbeam II laser, controlled by proprietary software Flexcontrol 3.4 (Bruker Daltonics). The Ultraflex was operated in reflectron positive (RP) ion mode, calibrated with a peptide calibration standard (Bruker Daltonics). For sample measurements 10,000 laser shots were accumulated at a laser frequency of 1000 Hz, using a complete sample random walk with 200 shots per raster spot. Tandem mass spectrometry (MALDI-TOF-MS/MS) was performed on the most abundant peaks of the mouse *N*-glycome via laser-induced dissociation. Fragmentation spectra were annotated using GlycoWorkbench (version 2.1) [32].

### Nano-liquid chromatography–mass spectrometry (Nano-LC-MS) analysis of IgG Fc-glycosylation

IgG was isolated from 2 μL mouse plasma and 150 μL peritoneal fluid using protein G-Sepharose affinity chromatography (GE Healthcare, Uppsala, Sweden), enzymatically digested with trypsin and finally analyzed by MS as described previously [33, 34]. Briefly, the tryptic IgG Fc *N*-glycopeptides were separated and analyzed on an Ultimate 3000 RSLC nanoLC system (Thermo Scientific; Sunnyvale, CA, USA). Separation was achieved on an Ascentis Express C18 nano-LC column (Supelco; Bellefonte, USA) conditioned with 900 nL/min 0.1 % TFA in water (mobile phase A) after which the following gradient of mobile phase A and 95 % ACN (mobile phase B) was applied; 0 min 3 % B, 2 min 6 % B, 4.5 min 18 % B, 5 min 30 % B, 7 min 30 % B, 8 min 1 % B and 11 min 1 % B. The MS detection was achieved using a quadrupole-TOF-MS (maXis Impact HD ultra-high resolution QTOF; Bruker Daltonics) [33, 34]. Sample ionization was accomplished using a tapered spray tip (internal diameter 20 μm) and a CaptiveSpray ion source (both from Bruker Daltonics) with a spray voltage set at 1300 V. A CaptiveSpray nanoBooster (Bruker Daltonics) was used with ACN saturated nitrogen to enhance sensitivity (0.2 bar). Drying temperature was set at 180 °C and drying gas-flow at 3 L/min (nitrogen 99.9990 %). Double and triple charged tryptic Fc glycopeptide signals were integrated and normalized to the subclass-specific total sum as described elsewhere [34].

### Data processing and statistical analysis

Automated MALDI-TOF-MS data processing was performed by using an in-house developed software named MassyTools [35]. MassyTools is released under the Apache 2.0 license and is freely available on GitHub (https://github.com/Tarskin/MassyTools). Briefly, spectra were exported as .txt file and recalibrated using a defined list of calibrant masses. Only spectra showing a signal-to-noise ratio (S/N) of 9 or above (root-mean-square) for at least five calibration masses were included for further analysis. The observed masses were then assigned to *N*-glycan structures according to monosaccharide composition, MALDI-TOF/TOF-MS/MS data, known biosynthetic pathways of glycans and literature [36–39]. Based on this assignment, a glycan feature list including 94 *N*-glycan peaks was generated (Supplementary Table S2) and used to proceed to the targeted data extraction of area-under-the-curve of each *N*-glycan profile. During data extraction, MassyTools dynamically determines the background around each isotopic peak and subtracts it from the area of each analyte peak intensity. Spectra exhibiting less than 45 % of the analyte area above S/N values of 9 were excluded from further analysis. Relative intensities of the defined set of 94 *N*-glycan peaks were calculated for each spectrum by setting the sum of area-under-the-curve values to 100 %. Furthermore, nineteen derived traits were calculated based on the compositional features (hexose = H; *N*-acetylhexosamine = N; fucose = F; *N*-acetylneuraminic acid = E or L for α2-6- and α2-3-linked variants respectively; *N*-glycolylneuraminic acid = Ge or Gl for α2-6- and α2-3-linked variants respectively). The formulae used to compute the derived glycosylation traits are described in supplementary Table S1. Statistical analyses and charts are based on biological replicates and were performed by using GraphPad Prism 6.0 software. For the comparison of glycosylation traits derived from total plasma *N*-glycome (TPNG) and total peritoneal fluid *N*-glycome (TPFG) of mice, paired t-test was used. A one-way analysis of variance (ANOVA) with post-hoc Tukey test was used for the multiple comparison of glycosylation traits in the different mice groups at baseline (0 h) and 24 h. Statistical comparison of the derived glycosylation traits between control and zymosan-induced peritonitis mice at the different time points was performed by two-way ANOVA with post-hoc Dunnett test. Data are presented as mean ± standard error of the mean (SEM). *p < 0.05; **p < 0.01; ***p < 0.001; ****p < 0.0001.

## Results

### Zymosan-induced peritonitis model and immunological profiles

**Fig. 1:**
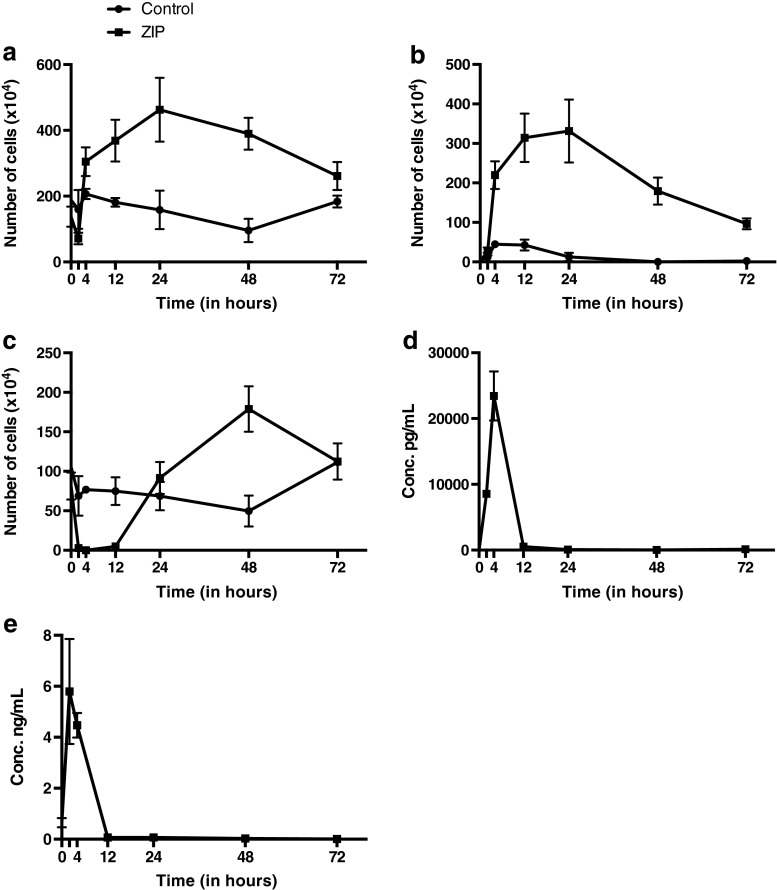
Recruitment of **a** leukocytes, **b** neutrophils and **c** monocytes becoming macrophages in(to) the peritoneal cavity of mice following zymosan-induced peritonitis (ZIP) or injection with PBS (control). Concentration of **d** IL-6 and **e** leukotriene B_4_ (LTB_4_) over time in the peritoneal fluid of ZIP mice

The zymosan-induced peritonitis model was used to investigate the influence of acute inflammation on TPNG and TPFG. To this end, female C57BL/6 mice were injected *i.p.* with zymosan. The mice were sacrificed at 0 h (to provide a baseline), 2, 4, 12, 24, 48 and 72 h and the plasma and peritoneal fluid samples were collected. Two independent experiments (1 and 2) were performed. As controls, a group of mice was injected *i.p.* with PBS (vehicle). The injection of zymosan resulted in a rapid, albeit transient elevation in the total number of peritoneal cells (Fig. 1a) [40]. The number of peritoneal neutrophils peaked at 24 h following zymosan injection (3.3 × 10^6^ cells) and then gradually declined (Fig. 1b). In contrast, a rapid decrease in the number of macrophages was observed during the first 12 h, probably due to disappearance of resident macrophages, followed by a rapid monocyte/macrophage influx into the inflamed cavity, peaking at 48 h after zymosan injection (1.8 × 10^6^ cells) (Fig. 1c) [26, 40, 41]. The acute inflammatory response resulting from the zymosan challenge was also reflected in a quick increase in the concentration of IL-6 returning to control levels after 12 h (Fig. 1d). A similar trend was observed for the concentration of leukotriene B_4_ (LTB_4_), a well-known eicosanoid mediating neutrophil chemotaxis (Fig. 1e) [26]. Importantly, injection with PBS (control mice) only had minimal effects on the number of total peritoneal cells, neutrophils and macrophages.

### Characterization of mouse total plasma *N*-glycome and total peritoneal fluid *N*-glycome

Initially, we characterized and compared the TPNG and the TPFG at baseline (0 h). Both glycomes were generated by performing a high-throughput release of *N*-glycans followed by chemical derivatization of sialic acids allowing stabilization and discrimination of α2-3−/α2-8- and α2-6-linked residues [20]. TPNG and TPFG samples were analyzed by MALDI-TOF(/TOF)-MS and the resulting data were processed using a new, in-house developed software for automated data processing of mass spectra of glycoconjugates [35]. In both glycomes, 94 peaks were assigned to glycan species according to their calculated compositions of monosaccharides and substituents (e.g. acetylation) and 19 derived glycosylation traits were calculated (Supplementary Tables S2 and S3). Importantly, the main glycan compositions and the key structural features thereof were confirmed by MALDI-TOF/TOF-MS/MS measurements (Supplementary Fig. S1).

**Fig. 2:**
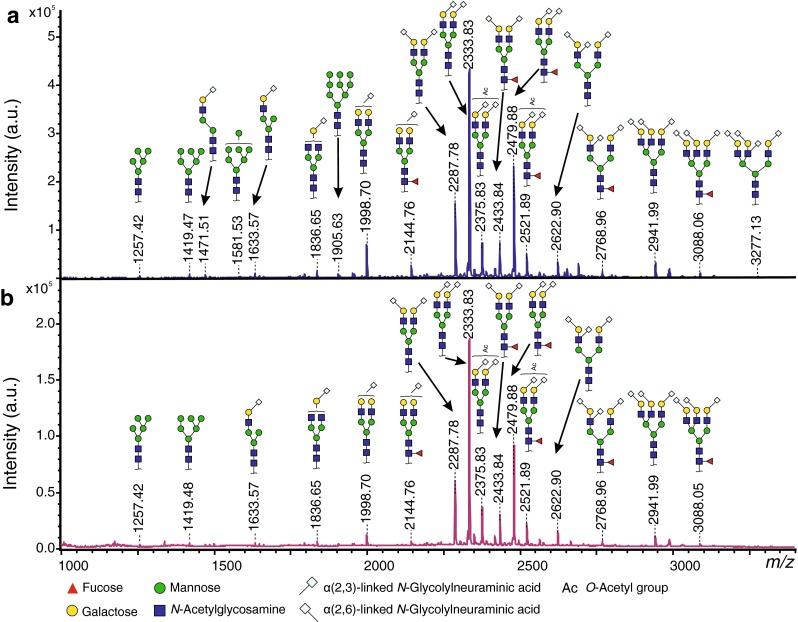
MALDI-TOF-MS spectra of mouse **a** TPNG and **b** TPFG at baseline (0 h). The proposed glycan structures are based on monosaccharide composition (Supplementary Table S2), literature as well as MS/MS data (Fig. 4 and Supplementary Fig. S1). Structures are depicted according to the CFG notation

**Fig. 3:**
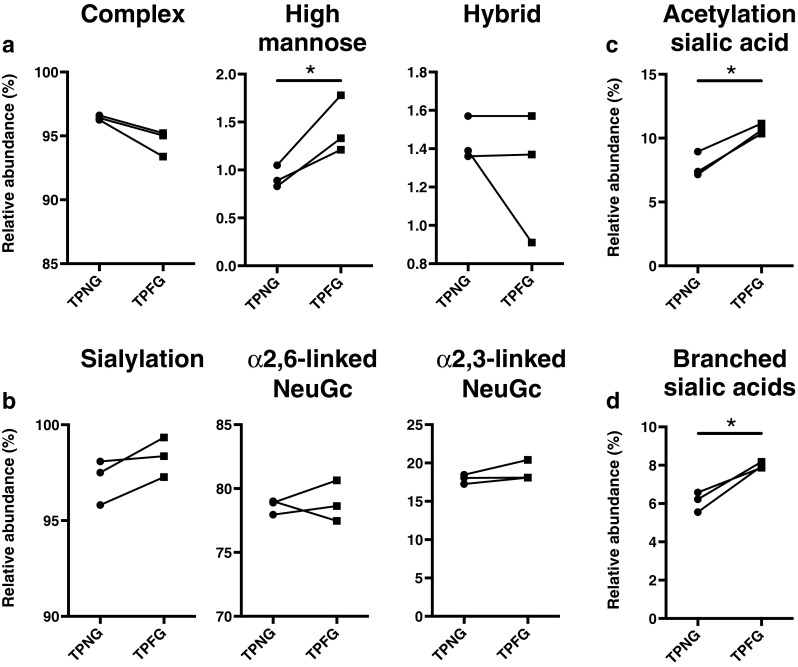
Comparison of the relative distribution of the *N*-glycan types and derived glycosylation traits in the TPNG and TPFG of mice at baseline (0 h). **a** Abundances of the three major types of *N*-glycans, i.e. *complex*, high mannose and hybrid *N*-glycans. **b** Levels of total sialylation (NeuAc & NeuGc) and linkage-specific (α2,6- vs α2,3-) NeuGc per antenna across all glycan species. As each *N*-glycan antenna can carry two sialic acid residues linked to both the terminal antenna galactose and the penultimate antenna GlcNAc residue, the level of sialylation (per antenna) can be over 100 %. Percentages of *N*-glycans containing **c** acetylated sialic acid and **d** branched sialic acid (NeuGc), i.e. a disialylated antenna sequence NeuGc-(α2,3)-Hex-[NeuGc-(α2,6)-]HexNAc, across all glycan species. Data were obtained in the first experiment of zymosan-induced peritonitis. Statistical analysis was performed for all glycan features using a paired t-test (*n* = *3*) (Supplementary Table S3). *p < 0.05

As illustrated in Fig. 2, the TPNG and TPFG exhibit highly similar glycosylation patterns. With more than 90 % of relative abundance, complex-type *N*-glycans predominate over high-mannose and hybrid-type species (Fig. 3 and Supplementary Table S3). Of note, a slightly higher abundance of high-mannose was observed in the TPFG (mean ± SEM 1.4 % ± 0.2 %) as compared to TPNG (0.9 % ± 0.1 %, *p* = 0.049, *n* = *3*, paired), which occur at the expense of complex-type *N*-glycans (Fig. 3 and Supplementary Table S3). The latter are mainly diantennary glycoforms (87.3 % ± 0.3 % and 85.6 % ± 0.7 % in TPNG and TPFG, respectively) followed by triantennary (9.1 % ± 0.4 % and 8.8 % ± 0.9 %, respectively), monoantennary (2.6 % ± 0.0 % and 3.8 % ± 0.3 %, respectively) and tetra-antennary species (<0.5 %) (Supplementary Table S3). Both glycomes exhibit a very high level of galactosylation per antenna (>95 %). Approximately 30 % of the glycans are fucosylated, mainly mono-fucosylated (Supplementary Table S3), with a fucose linked to the core GlcNAc residue as demonstrated by MS/MS experiment, showing the loss of the reducing end *N*-acetylglucosamine with fucose (−367.1 Da) from fucosylated precursor masses and the presence of a H3N2F1 fragment at *m*/*z* 1079.5 [M + Na]^+^(Supplementary Fig. S1). However, a very low level (<0.3 %) of di-fucosylated sialylated glycoforms (H4N4F2Gl1 and H5N4F2E1) was also detected, thus suggesting presence of antennary fucosylation (Supplementary Table S3). Fucosylation was detected in mono-, di-, and triantennary species but not in tetra-antennary glycans (data not shown). In both TPNG and TPFG, the total level of sialic acids (NeuAc and NeuGc) per antenna across all glycan species was over 97 %, with NeuAc and NeuGc accounting for less than 1 % and more than 96 %, respectively (Fig. 3 and Supplementary Table S3). Due to the linkage-specific derivatization of sialic acids, we could distinguish that around 79 % of the glycan antennae contain ethyl esterified NeuGc (Ge) indicating an α2-6-linkage, whereas lactonized NeuGc (Gl), indicating α2-3- or possibly α2-8-linkage, were present on only 18 % of glycan antennae (Fig. 3 and Supplementary Table S3).

**Fig. 4:**
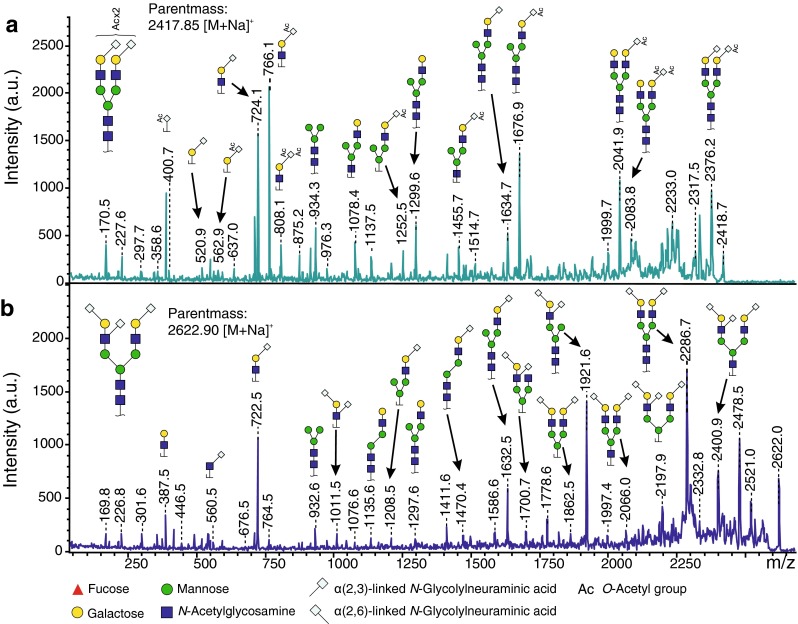
MALDI-TOF/TOF-MS/MS spectra of two *N*-glycans found in mouse TPNG and TPFG. **a** Disialylated diantennary complex-type *N*-glycan carrying two α2,6-linked NeuGc and two *O*-acetyl groups. **b** Trisialylated diantennary *N*-glycan exhibiting a monosialylated (α2,6-linked NeuGc) antenna and a disialylated antenna (NeuGc-(α2,3)-Hex-[NeuGc-(α2,6)-]HexNAc). Glycan fragments were assigned with the help of GlycoWorkbench version 2.1

The main glycan peak (H5N4Ge2) at *m*/*z* 2333.82 [M + Na]^+^, which accounts for 30 % of the total glycans, consists of a fully galactosylated diantennary complex-type *N*-glycan carrying two ethyl esterified (α2,6-linked) NeuGc (Ge) (Fig. 2, Supplementary Fig. S1 and Table S2). This glycan can be further extended by a core fucose (at *m*/*z* 2479.88) as well as one *O-*acetyl group (*O*Ac = 42.01 Da; *m*/*z* 2375.84), or two *O*Ac groups (+84.02 Da, *m*/*z* 2417.85). *O*-Acetylation is one of the most frequent modifications of sialic acids, in which an acetyl group is added to the hydroxyl group at C4, C7, C8, and/or C9 of the sialic acid by sialate *O*-acetyltransferases [42]. Accordingly, we only observed acetylation on sialylated glycans, indicating that acetyl modifications are probably located on NeuAc/NeuGc. This was also confirmed by the mono-*O*-acetylated sialylated antennae fragments at *m*/*z* 400.6 (Ge1Ac1) and 765.5 (H1N1Ge1Ac1) observed in the MALDI-TOF/TOF-MS/MS spectra of mono- and di-*O*-acetylated glycans H5N4Ge2Ac1, H5N4F1Ge2Ac1 and H5N4Ge2Ac2 (precursors at *m*/*z* 2375.84, 2521.89, 2417.85 [M + Na]^+^ respectively) (Fig. 4a and supplementary Fig. S1). Furthermore, the fragment at *m*/*z* 808.1 (H1N1Ge1Ac2), present in the MS/MS spectra of the di-acetylated glycan H5N4Ge2Ac2, indicated that both *O*Ac groups could be attached to a single NeuGc rather than one *O*Ac on each NeuGc (Fig. 4a). More generally, glycans from the peritoneal fluid exhibit a significantly higher level of sialic acid acetylation compared to plasma glycans (10.7 % ± 0.2 % vs 7.8 % ± 0.6 %, *p* = 0.016, *n* = *3*, paired) (Fig. 3 and Supplementary Table S3).

Another predominant glycoform (H5N4Ge1Gl1) at *m*/*z* 2287.78 [M + Na]^+^ is a fully galactosylated diantennary complex-type *N*-glycan carrying one ethyl esterified (α2-6-linked) *N*-glycolylneuraminic acid (Ge) and one lactonized (α2-3-linked) *N*-glycolylneuraminic acid (Gl) (Fig. 2, Supplementary Fig. S1 and Supplementary Table S2). This glycan can also be further extended in a non-exclusive manner by an acetyl group (e.g. H5N4Ge1Gl1Ac1 at *m*/*z* 2329.79), a fucose (H5N4F1Ge1Gl1 at *m*/*z* 2433.84 [M + Na]^+^), or by one or two (sialylated)-Gal-GlcNAc antennae giving rise to tri- and tetraantennary glycans (e.g. H6N5Ge1Gl2 and H7N6Ge1Gl2 at *m*/*z* 2941.99 and 3307.13 [M + Na]^+^, respectively) (Supplementary Table S2). Importantly, an additional NeuGc residue can be further attached to H5N4Ge1Gl1 leading to trisialylated diantennary complex-type *N*-glycans as described previously [36–39, 43]. The main trisialylated diantennary species at *m*/*z* 2622.90 [M + Na]^+^ carries a α2-6-linked *N*-glycolylneuraminic acid on one antenna and both α2-3-linked and α2,6-linked *N*-glycolylneuraminic acids on the galactose and the GlcNAc residues respectively of the other antenna (Fig. 2 and Supplementary Table S2). This structure was established by MALDI-TOF/TOF-MS/MS analysis, which produced fragments at *m*/*z* 560.5 (N1Ge1), at *m/z* 542.0 and *m*/*z* 1011.4 (H1N1Ge1Gl1) corresponding to the D ion NeuGc-(α2-6)-HexNAc and the ion NeuGc-(α2-3)-Hex-[NeuGc-(α2-6)-]HexNAc respectively (Fig. 4b) [36, 44]. Moreover, the absence of fragments at *m*/*z* 630 (Ge1Gl1) excludes the possibility of a NeuGc-(α2-8)-NeuGc motif (Fig. 4b). Of note, a higher level of sialylation of the GlcNAc residue, named branching sialylation, was observed in TPFG compared to TPNG (8.0 % ± 0.1 % vs 6.1 % ± 0.3 %, *p* = 0.028, *n* = *3*, paired) (Fig. 3 and Supplementary Table S3). In addition to these fully galactosylated and sialylated glycans, other related (truncated) glycoforms were detected such as H3N3Ge1, H4N3Ge1, H4N4Ge1 and H5N4Ge1 at *m*/*z* 1471.52, 1633.58, 1836.65 and 1998.70 [M + Na]^+^ (Fig. 2 and Supplementary Table S2).

To summarize, TPNG and TPFG exhibit highly related patterns that are characterized by a high level of fully galactosylated and sialylated complex-type *N*-glycans with or without core fucose. Sialic acids are predominantly NeuGc residues on which one or two *O*Ac groups can be attached. Moreover, we have identified α2-6-sialylation of the antennary GlcNAc of diantennary complex-type *N*-glycans that give rise to a disialylated antenna NeuGc-(α2-3)-Gal-[NeuGc-(α2-6)]-GlcNAc.

### Zymosan-induced peritonitis leads to rapid changes of fucosylation and sialylation in TPNG and TPFG

**Fig. 5:**
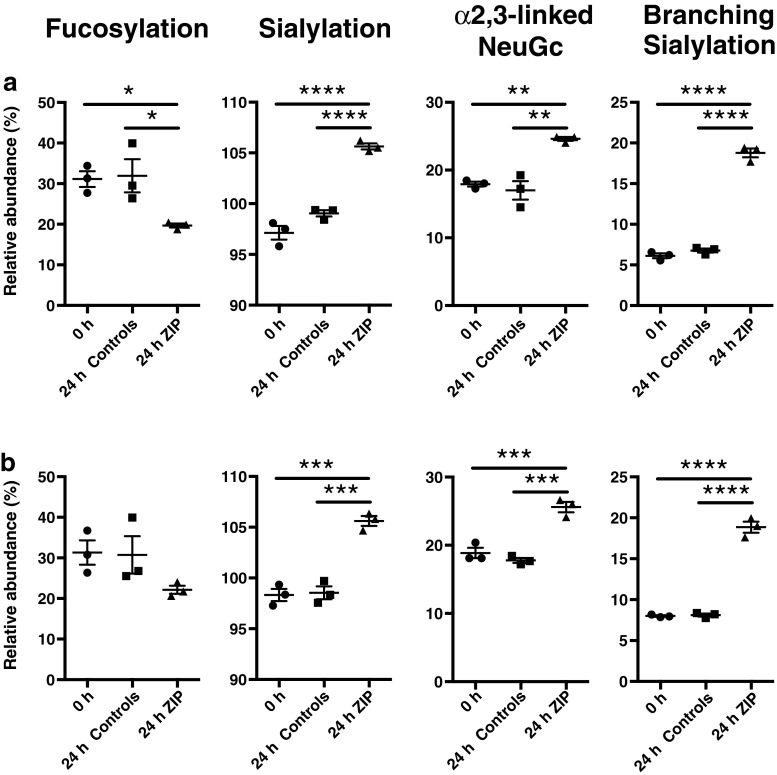
Comparison of selected derived glycosylation traits in the **a** TPNG and **b** TPFG of mice at time point 0 h and 24 h following challenge with zymosan (ZIP) or PBS (control). Description of the glycosylation features is provided in Fig. 3. Data belong to the first experiment of zymosan-induced peritonitis. Statistical analysis was performed for all glycosylation traits using a one-way analysis of variance (ANOVA) with post-hoc Tukey test (*n* = *3*) (Supplementary Table S4). *p < 0.05; **p < 0.01; ***p < 0.001; ****p < 0.0001

We next analyzed the changes in glycosylation occurring in TPNG and TPFG in response to zymosan or PBS (control) administration. To this end, we first compared the TPNG- and TPFG-derived glycosylation traits of zymosan-induced peritonitis mice 24 h after injection of the zymosan, the time point representing the peak of the inflammation (highest number of peritoneal leukocytes). Again, highly similar glycosylation patterns were observed between TPNG and TPFG (data not shown). We next compared the derived glycosylation traits of zymosan-induced peritonitis and control mice between 0 h (baseline) and 24 h post-injection. As shown in Fig. 5 and Supplementary Table S4, zymosan challenge induced several significant modifications of glycosylation in both TPNG and TPFG. Among the observed variations, the injection of zymosan resulted in a significant increase of triantennary complex-type *N*-glycans, which was not detected in control mice (Supplementary Table S4). Likewise, mice challenged with zymosan, but not controls, exhibit a significant decrease in the percentage of TPNG fucosylation after 24 h (Fig. 5a). A similar decrease of fucosylation level, albeit not statistically significant, was also observed in TPFG (Fig. 5b). In addition, zymosan-induced peritonitis led to a significant rise of the sialylation level in TPNG and TPFG, which is mainly due to a rise in the proportion of both α2-3-linked NeuGc and branching sialylation (Fig. 5). Likewise, although very low in abundance, both α2,3-linked and α2-6-linked NeuAc increased in TPNG and TPFG of mice 24 h after injection of the zymosan but not in control mice glycomes. In contrast, there was no clear evidence of changes in α2-6-linked *N*-glycolylneuraminic acid (Supplementary Table. S4). Finally, the acute zymosan-induced inflammation induced a significant decrease in the abundance of acetylated glycans (Supplementary Table S4). At the glycan level, the decrease of H5N4F1Ge2 and H5N4F1Ge2Ac1 as well as the increase of H4N4Ge2Gl1 were clearly identified as the main drivers of the changes in fucosylation, acetylation and sialylation (data not shown).

**Fig. 6:**
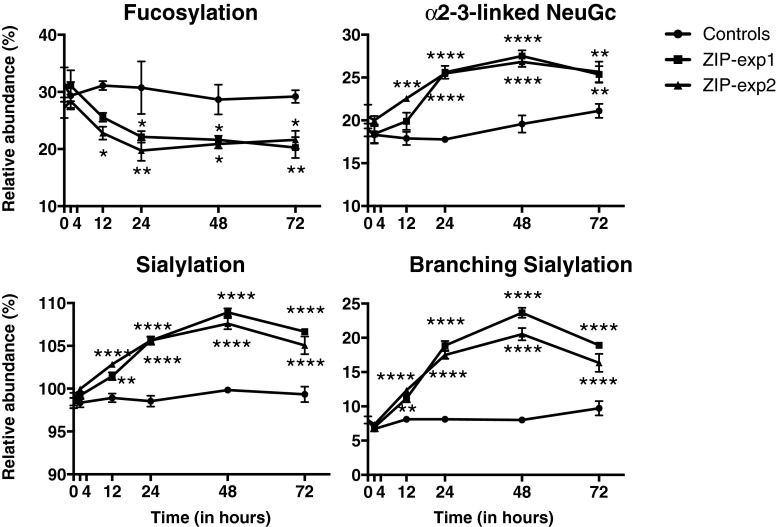
Longitudinal analysis of fucosylation, sialylation, α2-3-linked NeuGc and branched sialylation levels of the total peritoneal fluid *N*-glycome of mice following challenge with zymosan or PBS (control). Data belong to two independent zymosan-induced peritonitis experiments (ZIP-exp1 and ZIP-exp2). Statistical analysis was performed using a two-way analysis of variance (ANOVA) followed by a Dunnett test (*n* = *2–3*) (Supplementary Table S5). *p < 0.05; **p < 0.01; ***p < 0.001; ****p < 0.0001

In order to define the time point at which significant changes in glycosylation of zymosan-treated mice occur, we longitudinally analyzed selected glycosylation features including fucosylation, sialylation, α2-3-linked NeuGc and branching sialylation (Fig. 6, Supplementary Figure S2 and Table S5). We observed that the fucosylation level reached a minimum value at 24 h after zymosan challenge and then remained constant until at least the 72 h time point. In contrast, the sialylation-derived traits peaked at 48 h and then gradually declined. More importantly, as compared to control mice, significant changes in glycosylation of both TPNG and TPFG were observed in mice already at the 12 h time point, hence demonstrating unexpected velocity of the biological mechanisms involved. Of note, these results were successfully reproduced in a second independent zymosan-induced peritonitis experiment (Exp 2) (Fig. 6, Supplementary Figure S2 and Table S5).

### No changes in the glycosylation of plasma and peritoneal fluid IgG were observed following zymosan-induced peritonitis in mice

**Fig. 7:**
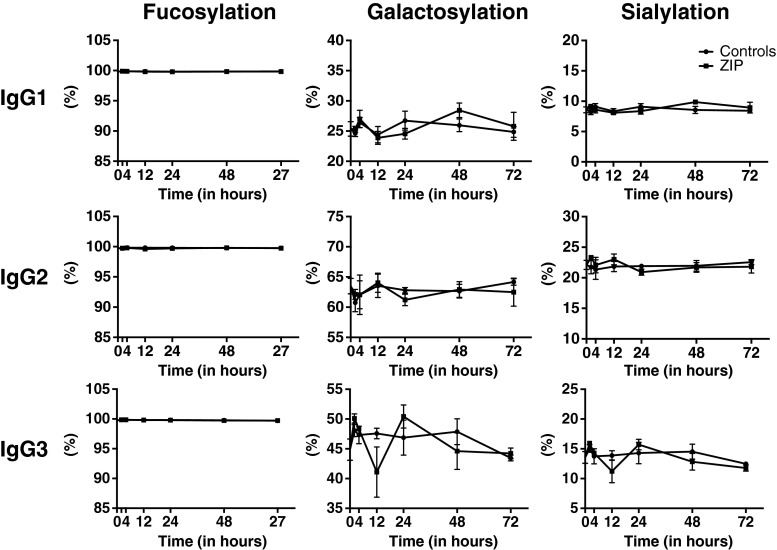
Longitudinal analysis of fucosylation, galactosylation and sialylation levels in IgG subclasses (IgG1, IgG2 and IgG3) isolated from the peritoneal fluid of mice in response to zymosan-induced peritonitis (ZIP) or PBS injection (controls). Results are depicted as mean ± SEM and were taken from the first experiment of zymosan-induced peritonitis (Supplementary Table S6)

It has now been clearly established that the Fc-linked glycosylation pattern of IgG, i.e. the level of sialylation, galactosylation and fucosylation, regulate the pro- or anti-inflammatory activity of antibodies through the modulation of binding to Fcγ-receptors, lectins (e.g. DC-SIGN) and proteins of the complement system [45–47]. Accordingly, changes in IgG glycosylation are associated with some physiological (e.g. pregnancy) and pathological conditions (e.g. rheumatoid arthritis) [17, 48, 49]. Recently, alterations of the IgG glycosylation pattern have been observed in patients within 24 h after heart surgery [18]. Therefore, we sought to determine whether acute inflammation in mice, due to zymosan-induced peritonitis, could also lead to a rapid reshaping of IgG glycosylation. To this end, the *N*-glycosylation of IgG was analyzed in a subclass-specific manner (IgG1, IgG2, IgG3) using a previously established LC-ESI-MS(/MS) method [16, 33, 34]. As observed for the glycomes, similar if not identical glycosylation features were found in IgG isolated from plasma and peritoneal fluid of mice (Supplementary Table S6). However, in contrast to TPNG and TPFG glycosylation, no significant differences in antibody galactosylation, sialylation and fucosylation could be detected between zymosan-induced peritonitis and controls, or across time points, for any of the IgG subclasses (Fig. 7, Supplementary Fig. S3 and Table S6).

## Discussion

Most of the *in vivo* immunological and medical research relies on mouse models, which are essential for elucidating mechanisms of (human) disease, as well as for translational research from bench to bedside. The murine zymosan-induced peritonitis is a widely used model to decipher the cellular and molecular mechanisms associated with acute inflammation and its controlled resolution. Among the large number of changes accompanying inflammation, significant variations in concentrations of many plasma and peritoneal proteins, known as acute-phase proteins (APP), have been observed [6]. As APP are glycosylated, many inflammatory conditions also result in alterations in total plasma protein glycosylation [13]. In this context, our study aimed at analyzing the dynamic changes in *N*-linked glycosylation of plasma and peritoneal fluid proteins occurring in mice during the course of self-resolving zymosan-induced acute inflammation.

The TPNG and TPFG were prepared and analyzed using a fast and reliable MALDI-TOF-MS–based analytical method as previously described [20, 21]. Among other advantages, this method enables the stabilization and selective derivatization of α2-6-linked and α2-3-linked sialic acids as well as the identification of glycan substituents such as *O*Ac groups. Structural analysis revealed a high similarity between TPNG and TPFG, which is in agreement with the important fluid exchange occurring between plasma and the peritoneal cavity [36]. In both glycomes, highly galactosylated and sialylated di- and triantennary complex type *N*-glycans predominated [36, 38]. In contrast to the NeuAc found in human, sialic acid residues in mouse TPNG and TPFG are almost exclusively NeuGc. Likewise, the high degree of sialylation observed in mice is in part due to the presence of disialylated antennae NeuGcα2-3-Galβ1-3-[NeuGcα2-6-]GlcNAc that, to our knowledge, has not been reported in humans. Of note, existence of such “branching sialylation” in mouse serum and liver tissue has been previously, rigorously characterized by NMR and MS/MS [36–39, 43]. Serotransferrin, haptoglobin, prothrombin and fibronectin have been identified as protein carriers for this disialylated epitope [36,39,50]. In addition to branching sialylation, we found that mouse *N*-glycomes contain *O*-acetylated sialic acids [38]. Although the meaning of this modification is far from clear, it has been shown that *O*-acetylation can block the recognition of sialic acids by intrinsic lectins (e.g. Siglecs) and modulate positively or negatively the interaction with microbial lectins [42]. Finally, fucosylation was detected in less than one-third of the glycans and MS/MS analyses demonstrated that fucose residues are mainly attached to the core GlcNAc of glycans. In other words, we were unable to demonstrate the presence of antennae fucose and Lewis-type antigens in mouse plasma and peritoneal fluid *N*-glycans, which is in agreement with the literature [36, 38, 51]. Of note, the sialyl Lewis^X^ epitope is present in human plasma *N*-glycans, carried by acute phase proteins and has often been associated with inflammation and disease severity [18, 20, 52–56].

We demonstrated that both mouse TPNG and TPFG are altered rapidly in response to zymosan-induced peritonitis. Especially, within 12 h, we observed a significant increase in the sialylation level together with a significant decrease of core fucosylation. Similar alterations in glycosylation have been previously reported in rat and mouse plasma proteins following turpentine oil-induced inflammation as well as in human total serum protein during the early course of sepsis and acute pancreatitis [55, 57–60].

We further showed that the rise in the level of mouse *N*-glycome sialylation after zymosan injection results from increases in α2-3-linked NeuGc and branching sialylation. Although this finding is novel, increase of branching sialylation of serum *N*-glycans has been previously observed in mice with colon cancer or during murine ovarian tumor progression stimulated by injections of pro-inflammatory drugs (thioglycolate or chlorite-oxidized oxyamylose) [36, 37]. The mouse sialyltransferase responsible for the addition of NeuGcα2-6 on the GlcNAc of the NeuGcα2-3-Galβ1-3-GlcNAc antenna has not been identified yet. However, enzymes with such activity have been previously characterized in the rat, fetal calf, rabbit and human liver, and placenta [61, 62]. These enzymes showed optimal activity with NeuGcα2-3-Galβ1-3-GlcNAc- acceptor structure but very low activity with the Galβ1,3-GlcNAc- sequence. If a similar enzyme exists in mouse liver, one can propose that the increase of branching sialylation is a direct consequence of the increase of α2-3-linked NeuGc. Notably, one possible enzyme candidate in mice is ST6GalNAc VI that exhibits activity towards sialyllactotetraosylceramide (NeuAcα2-3-Galβ1-3-GlcNAcβ1-3-Galβ1-4-Glcβ1-Cer) and whose mRNA transcript increases in the liver with inflammation [59, 63].

Several non-exclusive hypotheses can be proposed to explain the unexpectedly rapid changes in mouse glycomes following zymosan-induced peritonitis. First, the observed alterations in *N*-glycosylation could result from a rapid synthesis of cytokines/chemokines (e.g. IL-6) that induce quantitative changes of APP produced by the liver. This would imply a rapid synthesis of cytokines and APP with visible effects in the plasma and peritoneal fluid glycosylation. In line with this hypothesis, we showed that IL-6 peaked at 12 h post-injection, whereas the maximal glycosylation changes were observed between 24 and 48 h. Also, important changes in concentrations of glycosylated APP (e.g. haptoglobin, hemopexin, serum amyloid A) can already be detected in mice between 24 h and 48 h after induction of inflammation by *i.p.* injection of LPS [64–66]. In a second scenario, inflammatory mediators could directly affect the glycan composition of plasma proteins by regulating the biosynthetic pathways of glycosylation in the plasma protein producing cells. Accordingly, the rapid changes in rat plasma glycosylation in response to turpentine-oil inflammation occurred not only in positive APP (i.e. α1-acid glycoprotein, α2-macroglobulin), whose serum concentrations increase during inflammation, but also in negative APP (α1-inhibitor 3) and in non-acute phase proteins (α1-macroglobulin) produced by the liver [60]. Also, *in vitro* stimulation of hepatocytes and hepatoma cell lines with inflammatory stimuli (e.g. IL-6, TGF-β, TNF-α) leads to a direct modification of APP *N*-glycosylation [67]. Under the third hypothesis, changes in mouse *N*-glycosylation induced by zymosan challenge could be the consequence of a reshaping of plasma and peritoneal fluid protein glycosylation by circulating or membrane-bound glycosyltransferases and glycosidases, or following reversible endocytosis of plasma proteins by cells [60, 68]. The detection of a number of glycosyltransferase activities in systemic circulation supports this hypothesis [69]. The activity of blood glycosyltransferases, especially sialyltransferases, is increased during acute/chronic inflammation in mouse models and impacts the sialylation of IgG [70–72]. These extracellular enzymes may also be responsible for the extensive changes in human IgG glycome composition in the 24 h period following cardiac-surgery, which is unlikely to be the result of the production of new IgGs [18]. However, in this study, no significant change in mouse IgG glycosylation has been observed during zymosan-induced inflammation.

In summary, we demonstrated that rapid changes in mouse plasma and peritoneal fluid *N*-glycosylation occur during zymosan-induced peritonitis. Fucosylation, α2-3-linked NeuGc and branching sialylation represent markers of acute inflammation in mice. Future research should now focus on how these changes in glycosylation are caused, and how they may modulate the function of plasma and peritoneal acute phase proteins [13].

## Electronic supplementary material

ESM 1 (PDF 1.36 mb)

ESM 2 (PDF 400 kb)
