# Supplementary material for: Acute phase inflammation is characterized by rapid changes in plasma/peritoneal fluid N-glycosylation in mice
Source: Glycoconj J. 2016 Feb 29;33:457–70. doi: 10.1007/s10719-015-9648-9 (PMC4891370; doi:10.1007/s10719-015-9648-9)
Supplement: Supplementary file 1 — (PDF 1.36 mb) [file 10719_2015_9648_MOESM1_ESM.pdf]

Acute phase inflammation is characterized by rapid changes in plasma/peritoneal fluid *N*-glycosylation in mice

Yoann Rombouts[1,2,3,*], Hulda S. Jónasdóttir[1,2], Agnes L. Hipgrave Ederveen[1], Karli R. Reiding[1], Bas C. Jansen[1], Jona Freysdottir[4,5], Ingibjörg Hardardottir[4], Andreea Ioan-Facsinay[2], Martin Giera[1] and Manfred Wuhrer[1,6]

[1]Center for Proteomics and Metabolomics, Leiden University Medical Center, Leiden, The Netherlands; [2]Department of Rheumatology, Leiden University Medical Center, Leiden, The Netherlands; [3]Institut de pharmacologie et de Biologie Structurale, Université de Toulouse, CNRS, UPS, Toulouse, France [4]Faculty of Medicine, Biomedical Center, School of Health Sciences, University of Iceland, Reykjavik, Iceland; [5]Department of Immunology and Center for Rheumatology Research, Landspitali-The National University Hospital of Iceland, Reykjavik, Iceland; [6]Division of BioAnalytical Chemistry, VU University Amsterdam, The Netherlands.

*Corresponding author: Yoann Rombouts, yoann.rombouts@ipbs.fr, Tel. +33 (0)5 61 17 59 10

Supplementary Figures S1-S3

Supplementary Figure S1: MALDI-TOF/TOF-MS/MS spectra of major *N*-glycans from mouse TPNG and TPFG. Fragments were assigned with the help of GlycoWorkbench 2.1. Key: fucose (red triangle), mannose (green circle), GlcNAc (blue square), galactose (yellow circle), NeuGc (open diamond), *O*-acetylation (Ac). Linkage positions of sialic acid residues are indicated by differing angles: a2,3-linked NeuGc (\) and a2,6-linked NeuGc (/).

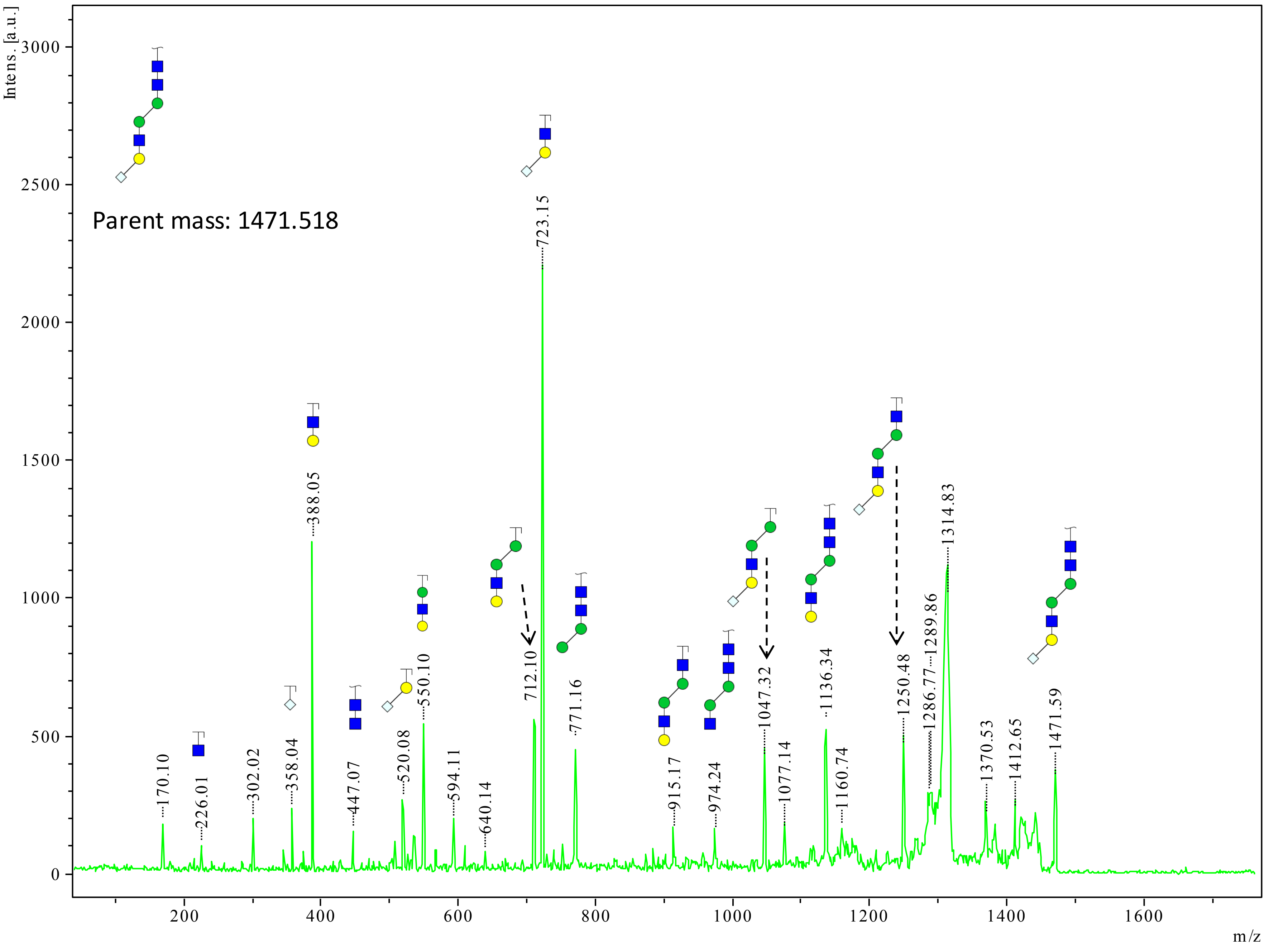
Parent mass: 1471.518
Intens. [a.u.]
m/z
170.10
226.01
302.02
358.04
388.05
447.07
520.08
550.10
594.11
640.14
712.10
723.15
771.16
915.17
974.24
1047.32
1077.14
1136.34
1160.74
1250.48
1286.77
1289.86
1314.83
1370.53
1412.65
1471.59

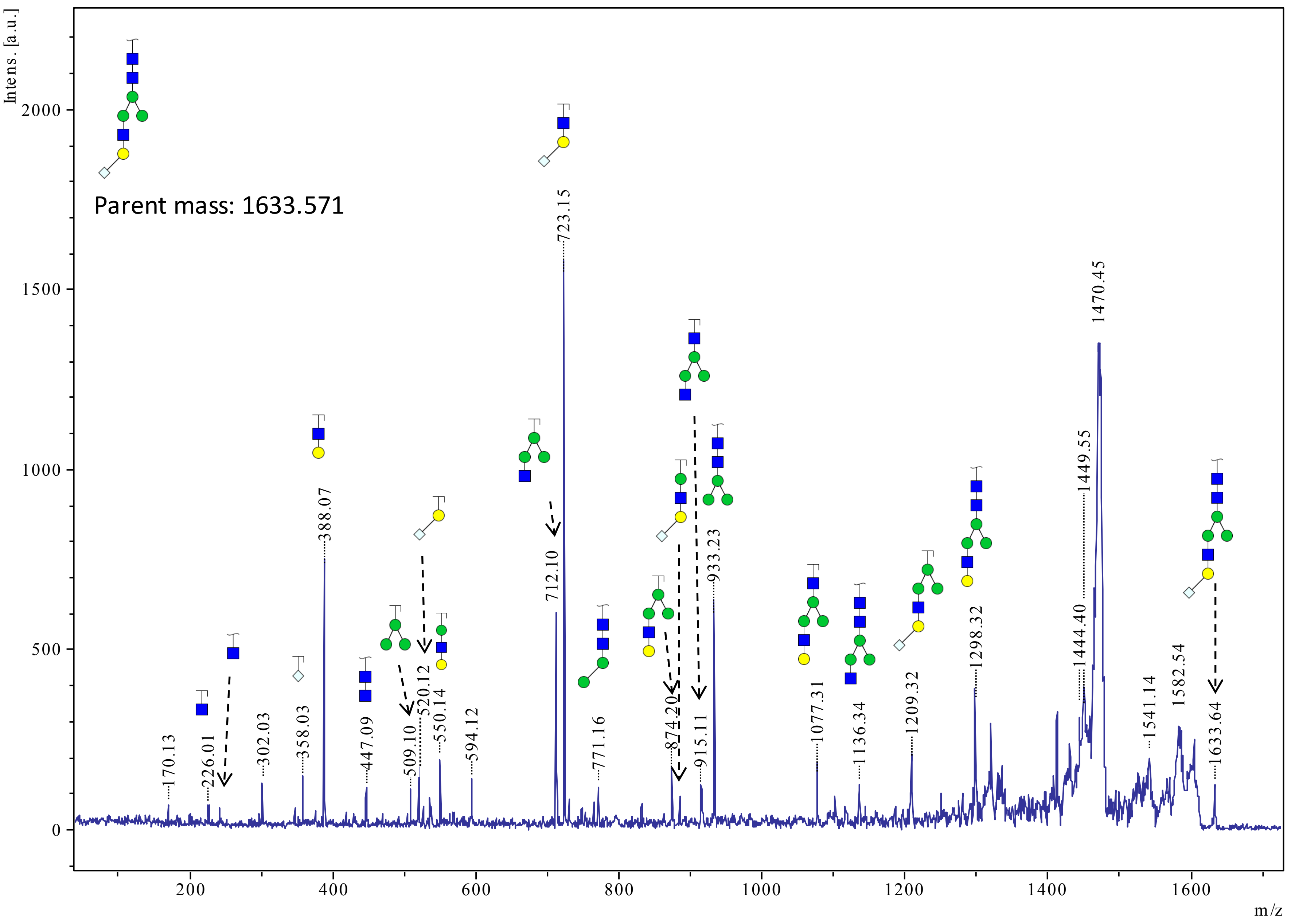
Intens. [a.u.]
Parent mass: 1633.571
2000
1500
1000
500
0
200
400
600
800
1000
1200
1400
1600
m/z
170.13
226.01
302.03
358.03
388.07
447.09
509.10
520.12
550.14
594.12
712.10
723.15
771.16
874.20
915.11
933.23
1077.31
1136.34
1209.32
1298.32
1444.40
1449.55
1470.45
1541.14
1582.54
1633.64

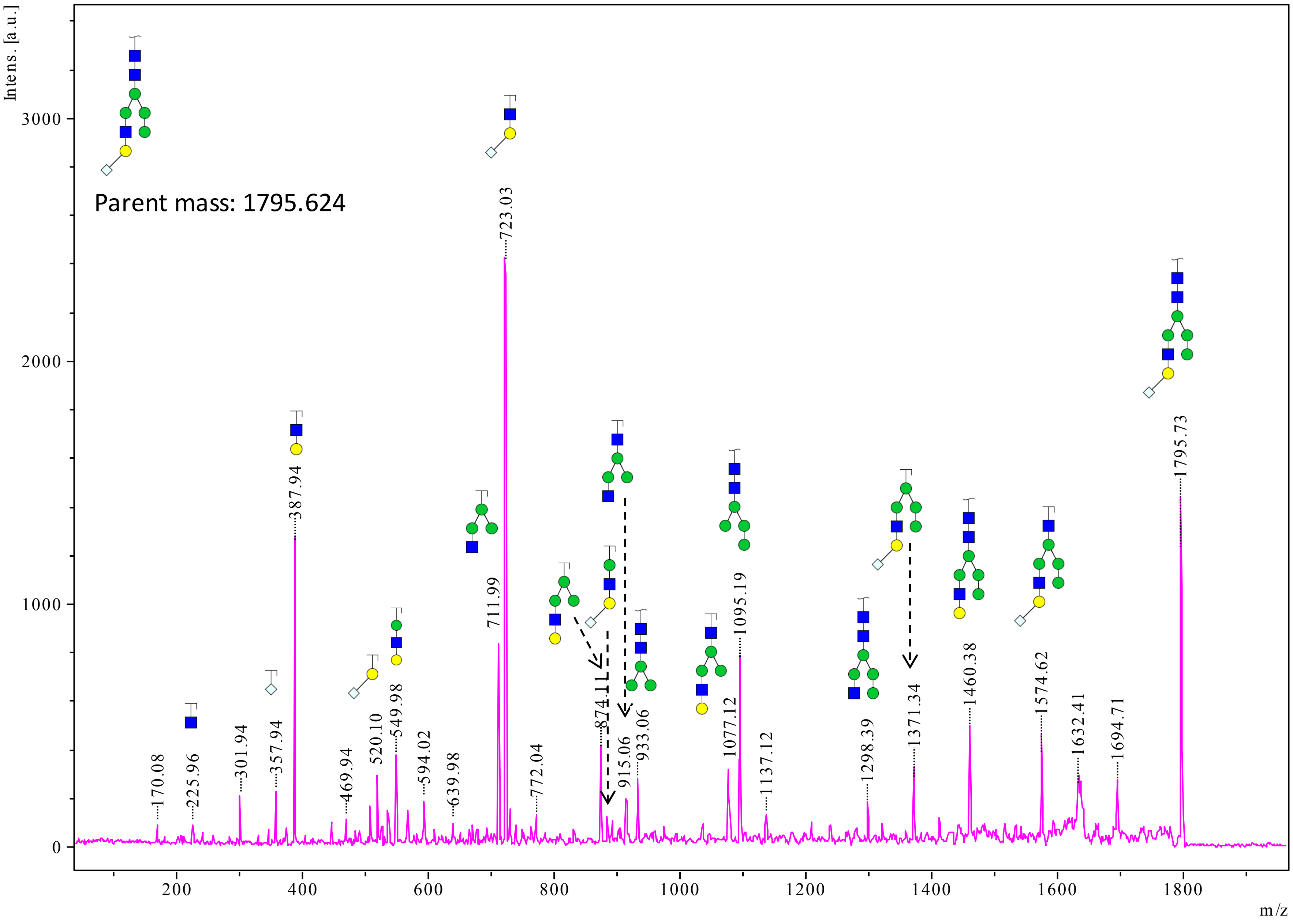

Intens. [a.u.]
Parent mass: 1795.624
3000
2000
1000
0
170.08
225.96
301.94
357.94
387.94
469.94
520.10
549.98
594.02
639.98
711.99
723.03
772.04
874.11
915.06
933.06
1077.12
1095.19
1137.12
1298.39
1371.34
1460.38
1574.62
1632.41
1694.71
1795.73
200
400
600
800
1000
1200
1400
1600
1800
m/z

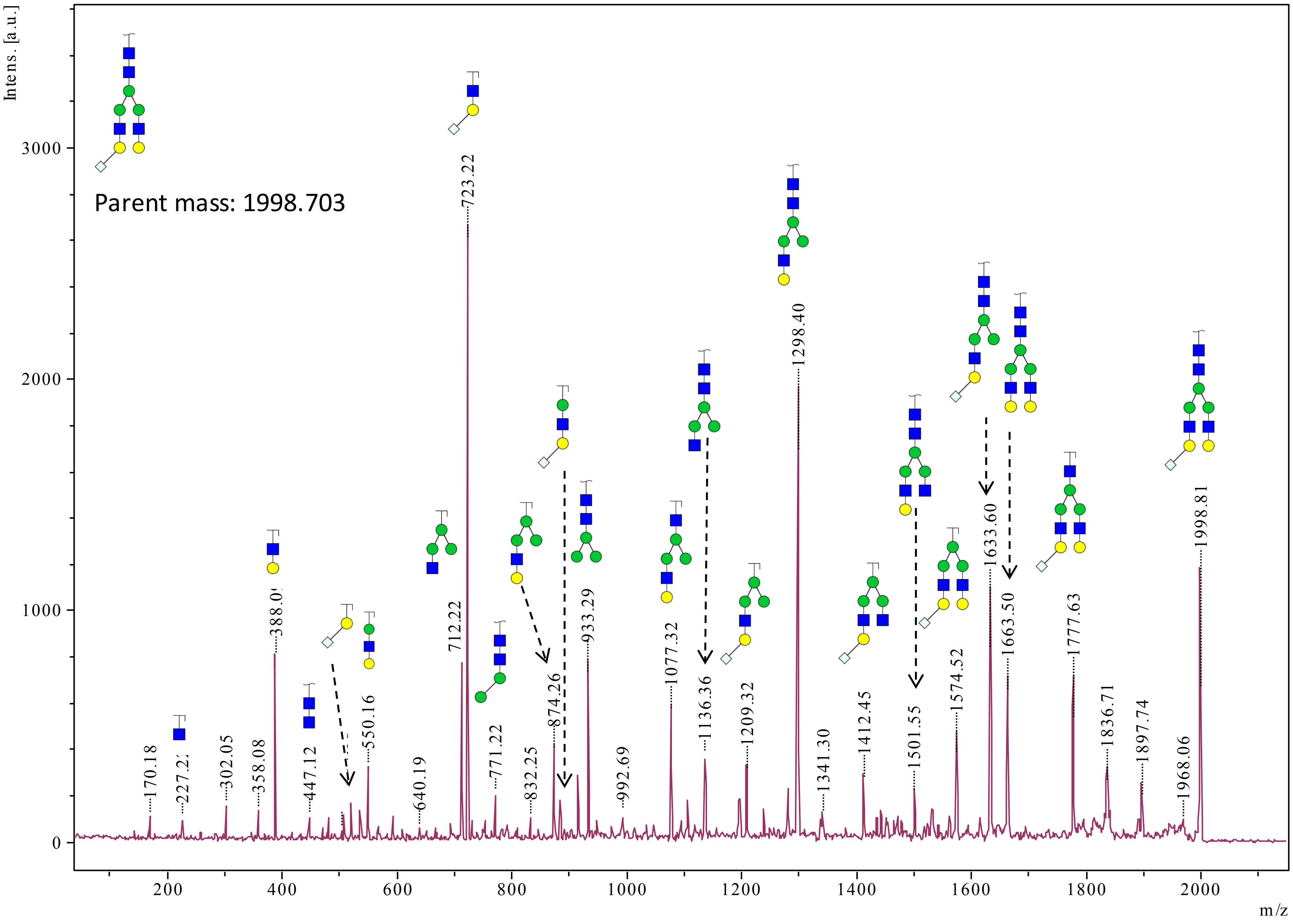

Intens. [a.u.]
3000
2000
1000
0
Parent mass: 1998.703
170.18
227.2
302.05
358.08
388.0
447.12
550.16
640.19
712.22
723.22
771.22
832.25
874.26
933.29
992.69
1077.32
1136.36
1209.32
1298.40
1341.30
1412.45
1501.55
1574.52
1633.60
1663.50
1777.63
1836.71
1897.74
1968.06
1998.81
200
400
600
800
1000
1200
1400
1600
1800
2000
m/z

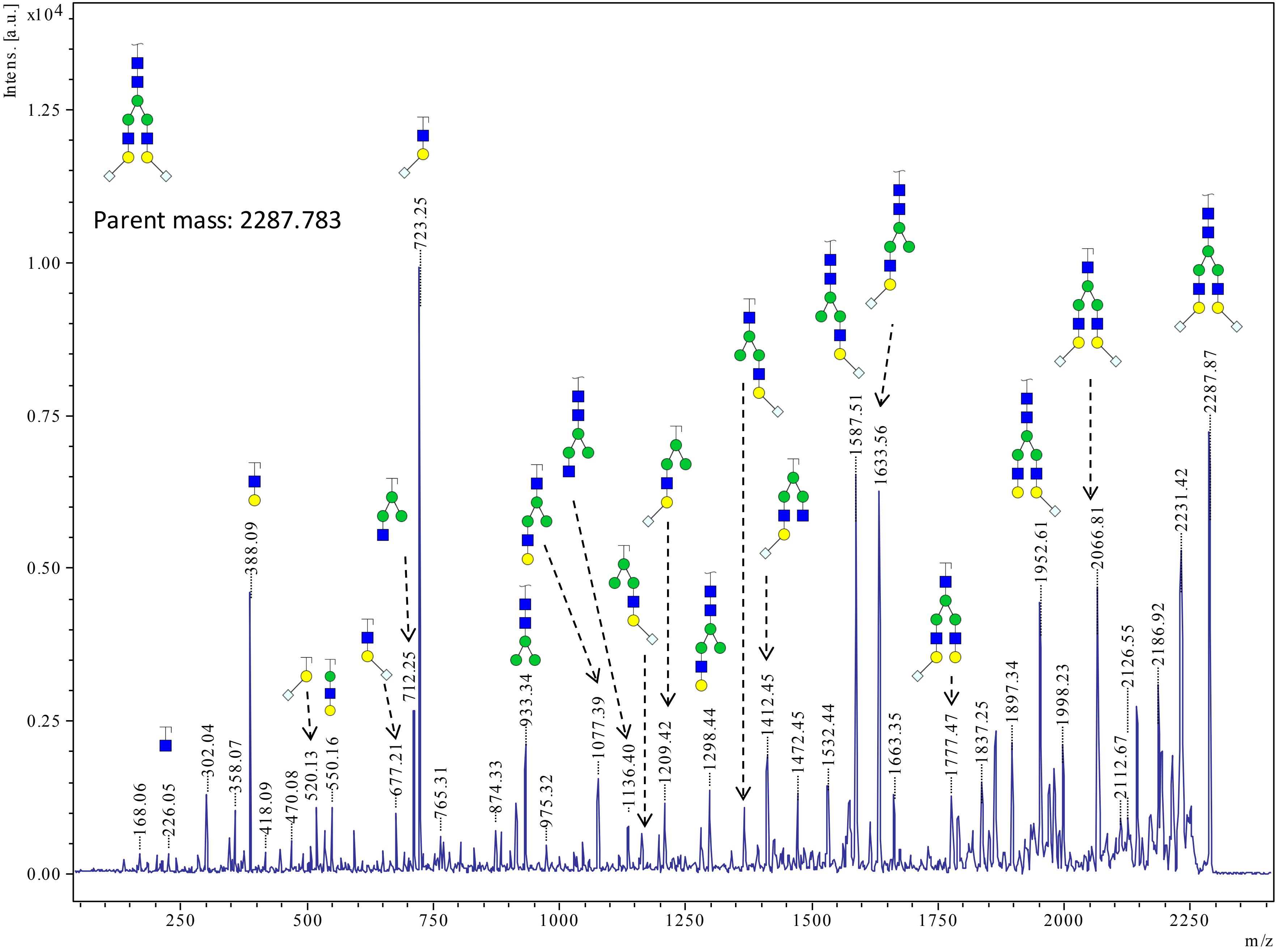

Parent mass: 2287.783
x10^4
Intens. [a.u.]
1.25
1.00
0.75
0.50
0.25
0.00
m/z
250
500
750
1000
1250
1500
1750
2000
2250
168.06
226.05
302.04
358.07
388.09
418.09
470.08
520.13
550.16
677.21
712.25
723.25
765.31
874.33
933.34
975.32
1077.39
1136.40
1209.42
1298.44
1412.45
1472.45
1532.44
1587.51
1633.56
1663.35
1777.47
1837.25
1897.34
1952.61
1998.23
2066.81
2112.67
2126.55
2186.92
2231.42
2287.87

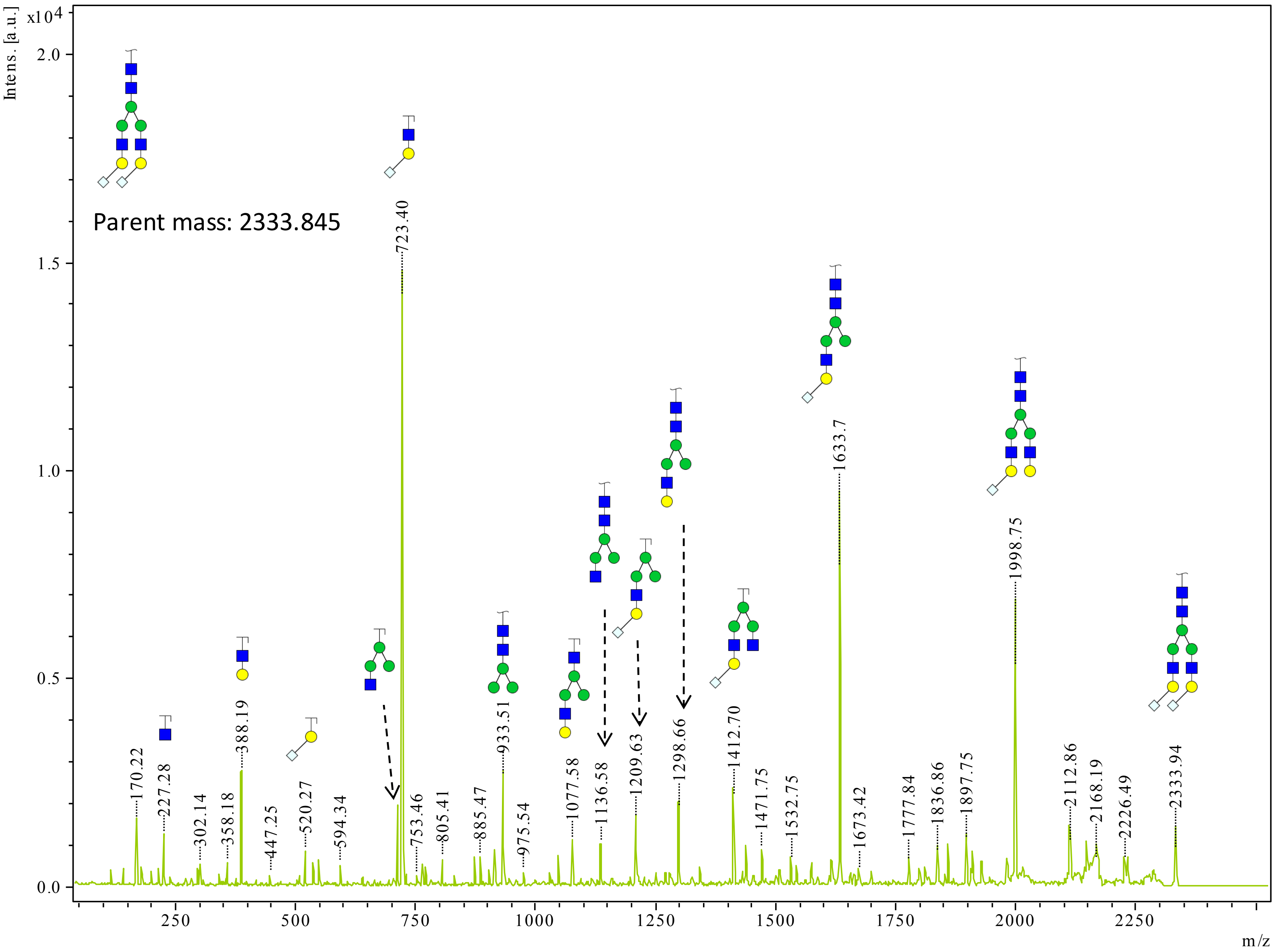

Parent mass: 2333.845
Intens. [a.u.]
x10^4
2.0
1.5
1.0
0.5
0.0
723.40
1633.7
1998.75
170.22
227.28
302.14
358.18
388.19
447.25
520.27
594.34
753.46
805.41
885.47
933.51
975.54
1077.58
1136.58
1209.63
1298.66
1412.70
1471.75
1532.75
1673.42
1777.84
1836.86
1897.75
2112.86
2168.19
2226.49
2333.94
250
500
750
1000
1250
1500
1750
2000
2250
m/z

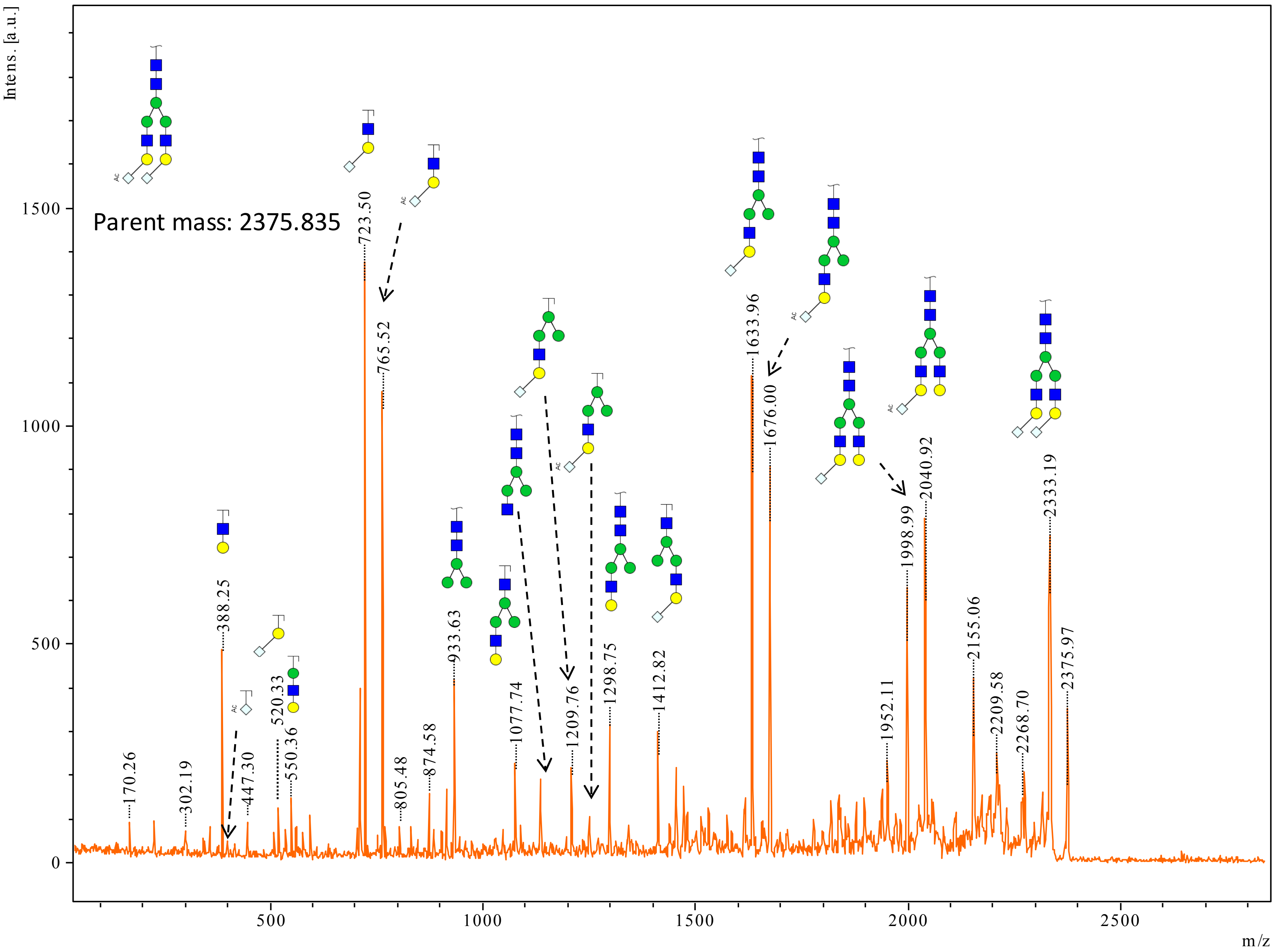

Intens. [a.u.]
m/z
Parent mass: 2375.835
170.26
302.19
388.25
447.30
520.33
550.36
723.50
765.52
805.48
874.58
933.63
1077.74
1209.76
1298.75
1412.82
1633.96
1676.00
1952.11
1998.99
2040.92
2155.06
2209.58
2268.70
2333.19
2375.97

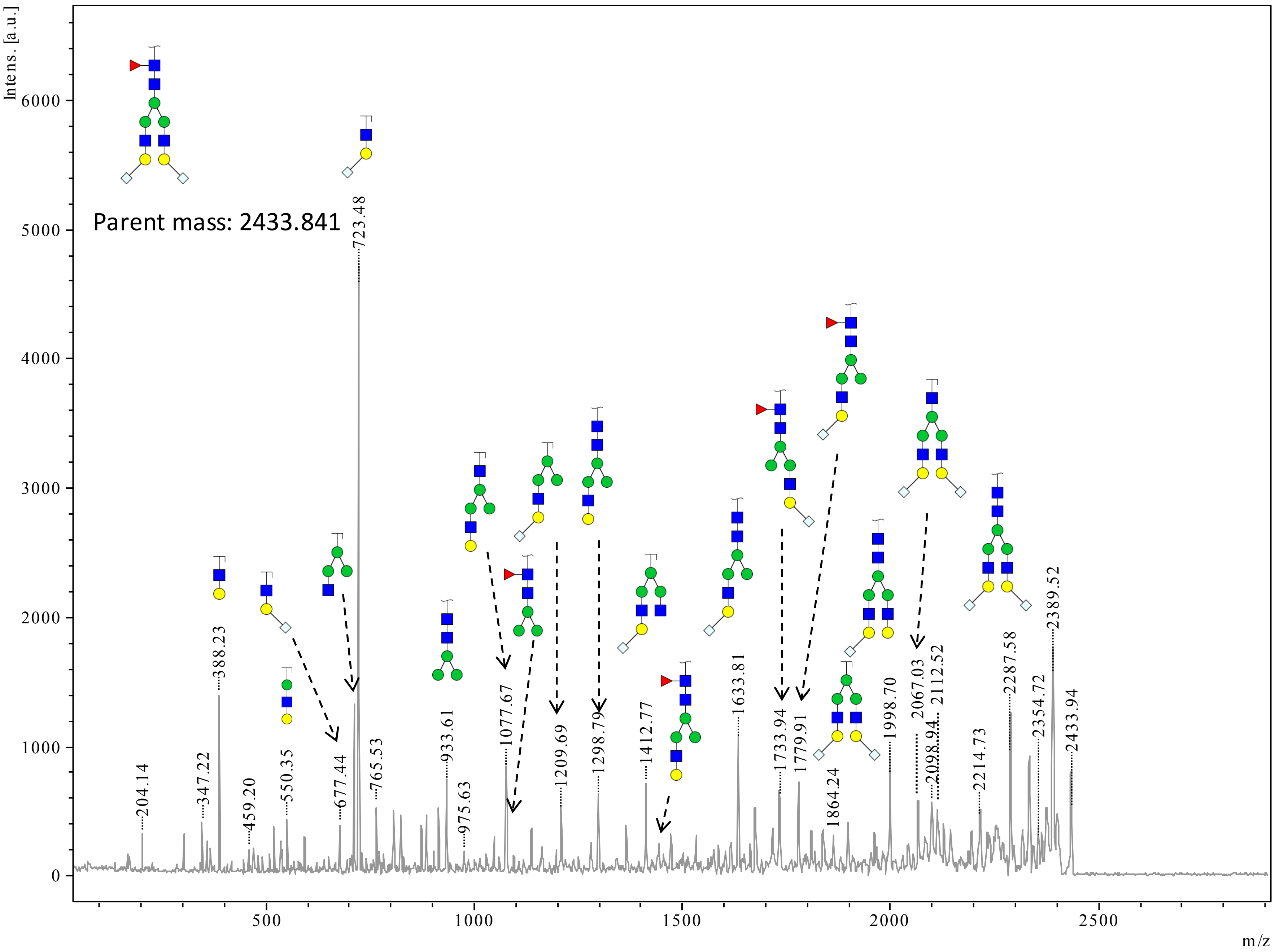

Intens. [a.u.]
Parent mass: 2433.841
6000
5000
4000
3000
2000
1000
0
500
1000
1500
2000
2500
m/z
204.14
347.22
388.23
459.20
550.35
677.44
723.48
765.53
933.61
975.63
1077.67
1209.69
1298.79
1412.77
1633.81
1733.94
1779.91
1864.24
1998.70
2067.03
2098.94
2112.52
2214.73
2287.58
2354.72
2389.52
2433.94

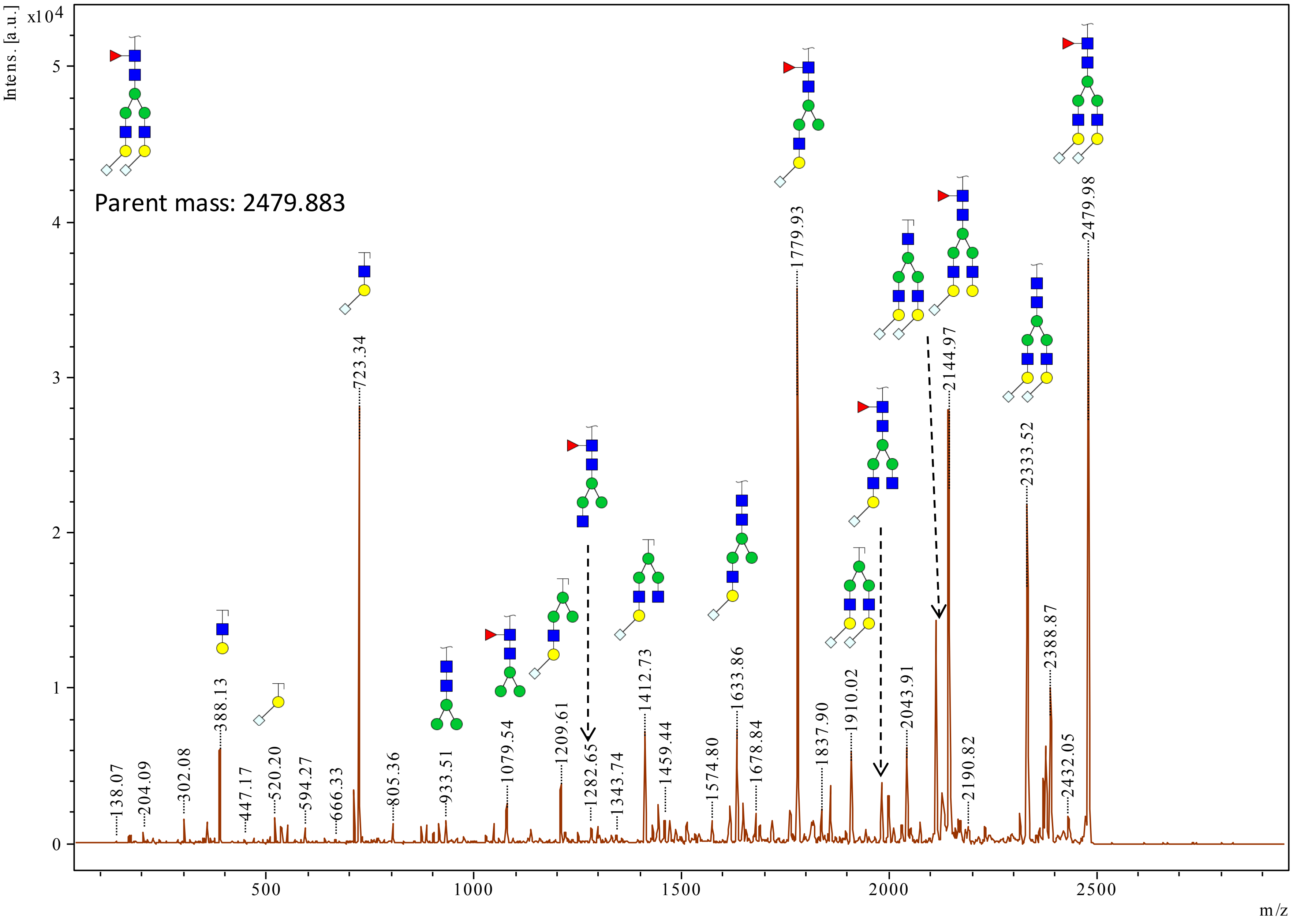

Intens. [a.u.]
x10^4
Parent mass: 2479.883
138.07
204.09
302.08
388.13
447.17
520.20
594.27
666.33
723.34
805.36
933.51
1079.54
1209.61
1282.65
1343.74
1412.73
1459.44
1574.80
1633.86
1678.84
1779.93
1837.90
1910.02
2043.91
2144.97
2190.82
2333.52
2388.87
2432.05
2479.98
m/z

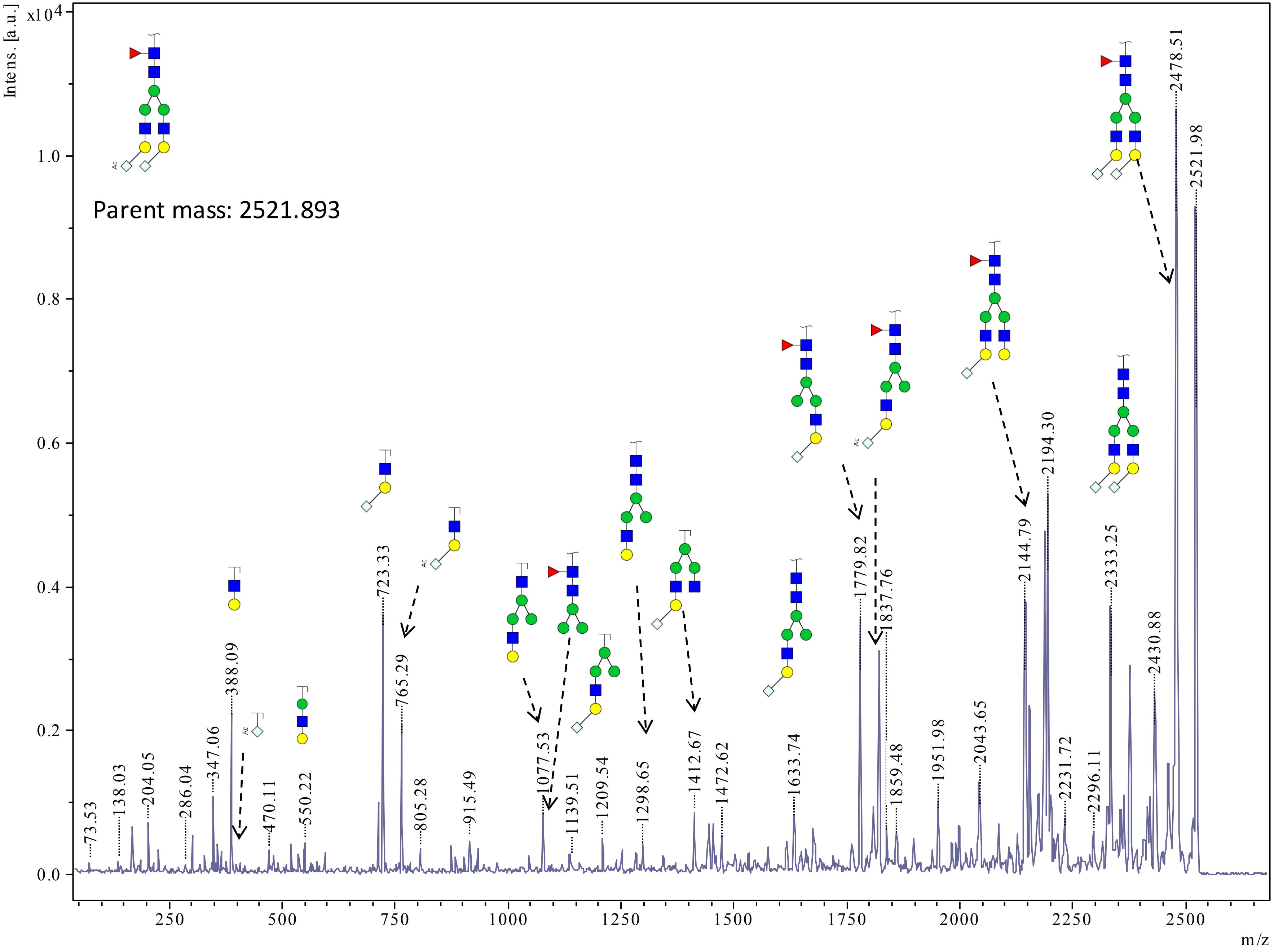

Intens. [a.u.]
x10^4
Parent mass: 2521.893
m/z
Ac
73.53
138.03
204.05
286.04
347.06
388.09
470.11
550.22
723.33
765.29
805.28
915.49
1077.53
1139.51
1209.54
1298.65
1412.67
1472.62
1633.74
1779.82
1837.76
1859.48
1951.98
2043.65
2144.79
2194.30
2231.72
2296.11
2333.25
2430.88
2478.51
2521.98

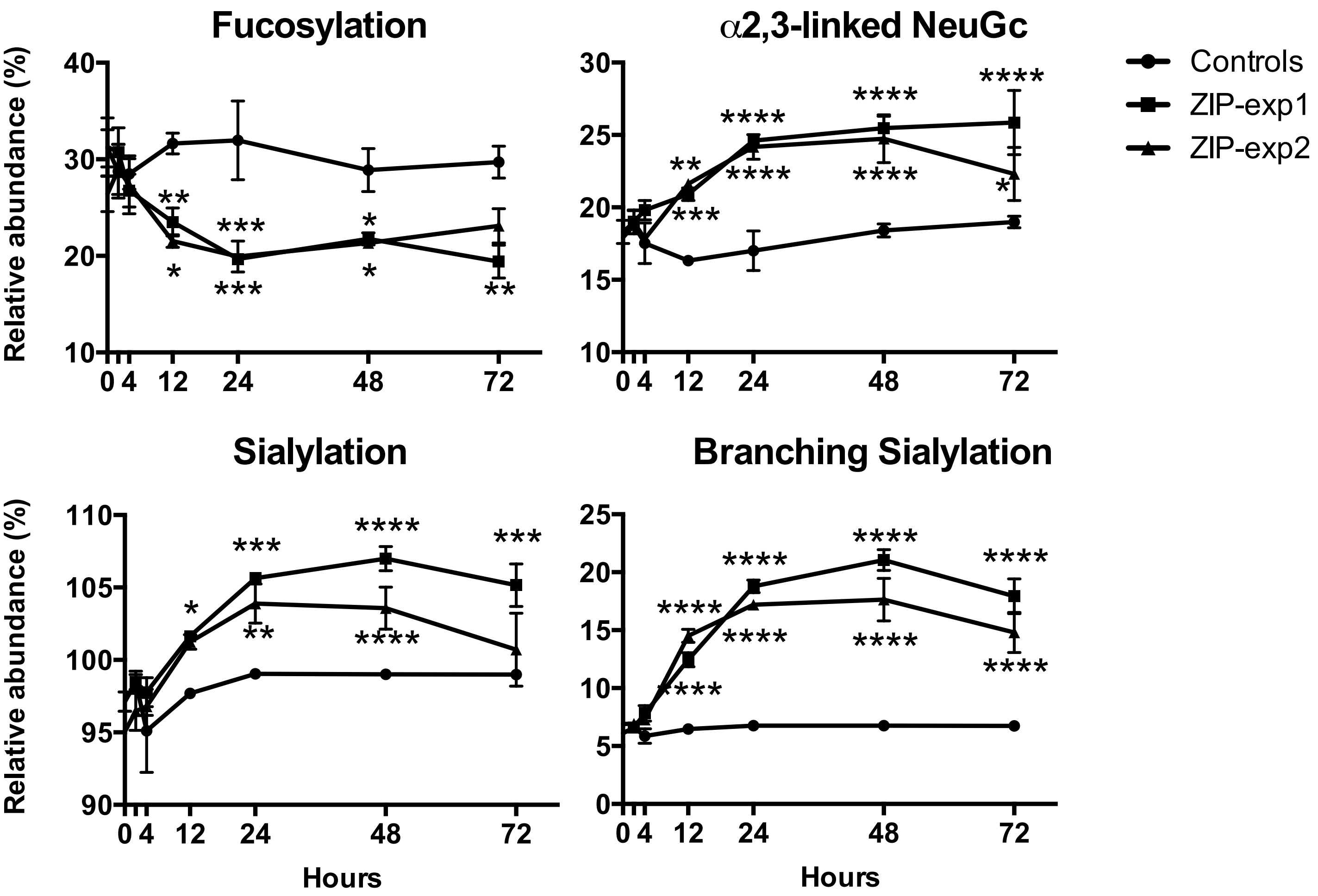

Supplementary Figure S2: Longitudinal analysis of fucosylation, sialylation, α2,3-linked NeuGc and branched sialylation levels of the total plasma *N*-glycome of mice following challenge with zymosan or PBS (control). Data belong to two independant zymosan-induced peritonitis experiments (ZIP-exp1 and ZIP-exp2). Statistical analysis was performed using a two-way analysis of variance (ANOVA) following by a Dunnett test (*n=3*) (Supplemental Table S5). **P* < 0.05; ***P* < 0.01; ****P* < 0.001; *****P* < 0.0001.

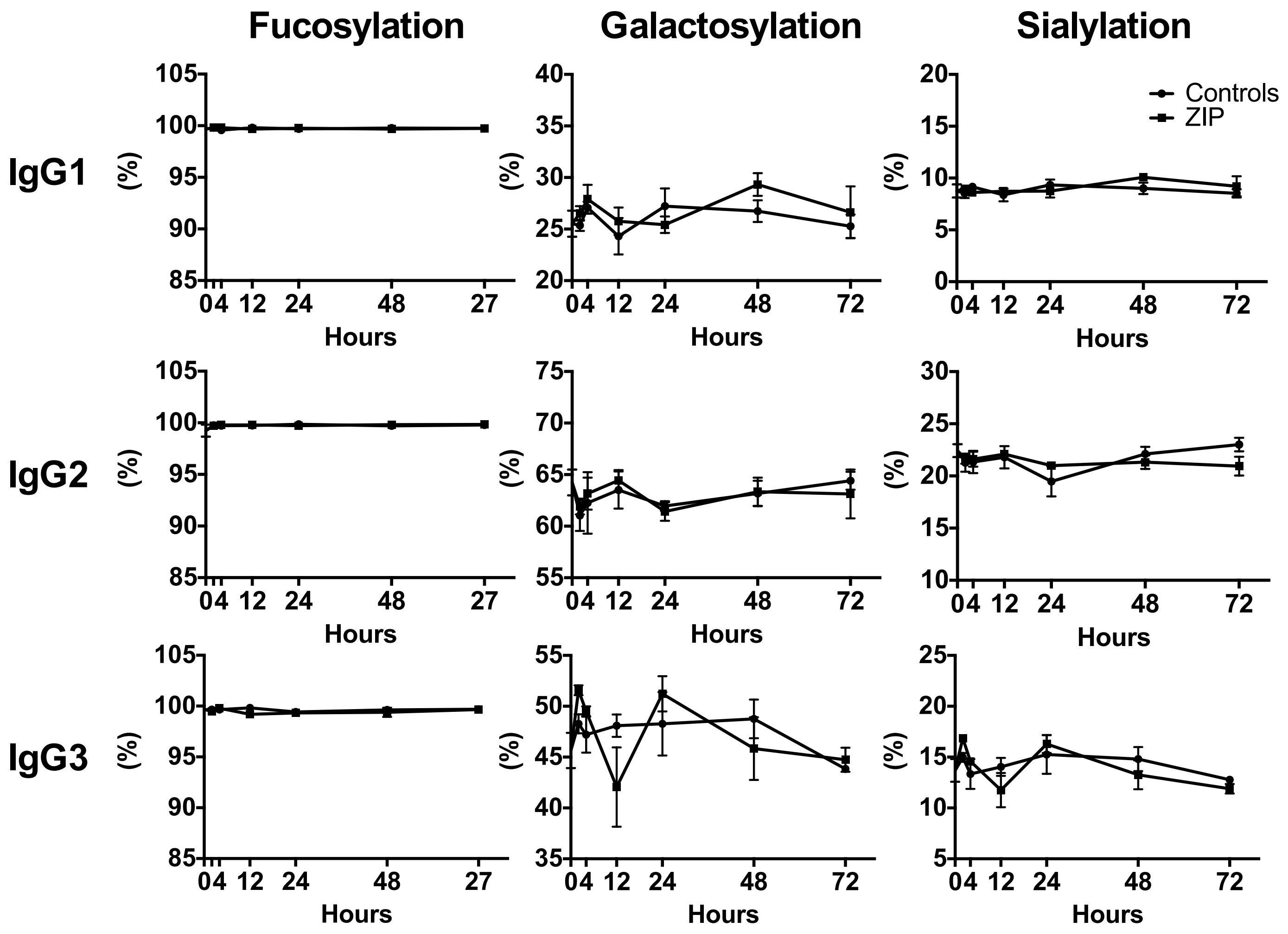

Supplementary Figure S3: Longitudinal analysis of fucosylation, galactosylation and sialylation levels in IgG subclasses (IgG1, IgG2 and IgG3) isolated from the plasma of mice following zymosan-induced peritonitis (ZIP) or PBS injection (controls). Results are depicted as mean ± s.e.m and were taken from the first experiment of zymosan-induced peritonitis.
