# Supplementary material for: Acute phase inflammation is characterized by rapid changes in plasma/peritoneal fluid N-glycosylation in mice
Source: Glycoconj J. 2016 Feb 29;33:457–70. doi: 10.1007/s10719-015-9648-9 (PMC4891370; doi:10.1007/s10719-015-9648-9)
Supplement: Supplementary file 2 — (PDF 400 kb) [file 10719_2015_9648_MOESM2_ESM.pdf]

# Acute phase inflammation is characterized by rapid changes in plasma/peritoneal fluid *N*-glycosylation in mice

Yoann Rombouts[1,2,3,*], Hulda S. Jónasdóttir[1,2], Agnes L. Hipgrave Ederveen[1], Karli R. Reiding[1], Bas C. Jansen[1], Jona Freysdottir[4,5], Ingibjörg Hardardottir[4], Andreea Ioan-Facsinay[2], Martin Giera[1] and Manfred Wuhrer[1,6]

[1]Center for Proteomics and Metabolomics, Leiden University Medical Center, Leiden, The Netherlands; [2]Department of Rheumatology, Leiden University Medical Center, Leiden, The Netherlands; [3]Institut de pharmacologie et de Biologie Structurale, Université de Toulouse, CNRS, UPS, Toulouse, France [4]Faculty of Medicine, Biomedical Center, School of Health Sciences, University of Iceland, Reykjavik, Iceland; [5]Department of Immunology and Center for Rheumatology Research, Landspitali-The National University Hospital of Iceland, Reykjavik, Iceland; [6]Division of BioAnalytical Chemistry, VU University Amsterdam, The Netherlands.

*Corresponding author: Yoann Rombouts, yoann.rombouts@ipbs.fr, Tel. +33 (0)5 61 17 59 10

Supplementary Tables S1-S6

Supplementary Table S1: Description of the formula for computing the derived glycosylation traits based on the identified glycans described in supplementary Table S2. H = hexose; N = *N*-acetylhexosamine; F = fucose; E = α2,6-linked *N*-acetylneuraminic acid; L= α2,3-linked *N*-acetylneuraminic acid; Ge = α2,6-linked *N*-glycolylneuraminic acid; Gl= α2,3-linked *N*-glycolylneuraminic acid; Ac = acetyl group.

| Abreviation | Derived glycosylation traits | Formula |
|---|---|---|
| T | All structures | H5N2 + H3N3F1 + H4N3 + H3N4 + H6N2 + H4N3F1 + H3N3Ge1 + H3N4F1 + H4N4 + H3N3Ge1Ac1 + H7N2 + H4N3Gl1 + H4N3E1 + H4N3Ge1 + H4N4F1 + H5N4 + H4N3Ge1Ac1 + H3N5F1 + H4N5 + H8N2 + H5N3Gl1 + H3N3Ge1Gl1 + H4N3F1Ge1 + H4N4Gl1 + H5N3Ge1 + H5N4F1 + H4N4Ge1 + H4N5F1 + H4N4Ge1Ac1 + H9N2 + H6N3Gl1 + H4N3Ge1Gl1 + H4N4F1Gl1 + H5N4Gl1 + H6N3Ge1 + H4N4F1Ge1 + H5N4Ge1 + H5N4Ge1Ac1 + H4N5Ge1Ac1 + H4N4F2Gl1 + H5N3Ge1Gl1 + H5N4F1Gl1 + H5N4F1Ge1 + H5N3Ge2Ac1 + H5N4Gl2 + H5N4E1Gl1 + H5N4L1Ge1 + H5N4F2E1 + H5N4Ge1Gl1 + H6N5Gl1 + H5N4Ge1Gl1Ac1 + H5N4Ge2 + H5N4Ge2Ac1 + H4N4Ge1Gl2 + H5N4Ge2Ac2 + H5N4F1Ge1Gl1 + H5N4F1L1Ge1Ac1 + H5N4F1Ge2 + H5N4F1Ge2Ac1 + H5N4F1Ge2Ac2 + H5N4Ge1Gl2 + H5N4L1Ge2 + H5N4Ge2Gl1 + H6N5Ge1Gl1 + H5N4Ge2Gl1Ac1 + H5N4Ge2Gl1Ac2 + H5N4F1Ge1Gl2 + H5N4F1Ge2Gl1 + H6N5F1Ge1Gl1 + H5N4F1Ge2Gl1Ac1 + H5N4F1Ge2Gl1Ac2 + H5N4Ge2Gl2 + H6N5Ge1Gl2 + H6N5Ge1Gl2Ac1 + H6N5Ge2Gl1 + H5N4F1Ge1Gl3 + H6N5Ge1Gl2Ac2 + H6N5Ge2Gl1Ac1 + H6N5Ge3 + H5N4F1Ge2Gl2 + H6N5Ge1Gl2Ac3 + H6N5Ge2Gl1Ac2 + H6N5F1Ge1Gl2 + H6N5F1Ge1Gl2Ac1 + H6N5F1Ge2Gl1 + H6N5F1Ge1Gl2Ac2 + H6N5F1Ge1Gl2Ac3 + H6N5Ge1Gl3 + H6N5Ge2Gl2 + H7N6Ge1Gl2 + H6N5Ge2Gl2Ac1 + H6N5F1Ge1Gl3 + H6N5F1Ge2Gl2 + H7N6Ge1Gl3 |
| M | High mannose glycans | H5N2 + H6N2 + H7N2 + H8N2 + H9N2 / T |
| H | Hybrid glycans | H5N3Gl1 + H5N3Ge1 + H6N3Gl1 + H6N3Ge1 + H5N3Ge1Gl1 + H5N3Ge2Ac1 / T |
| C | Complex glycans | H3N4 + H3N4F1 + H4N4 + H4N4F1 + H5N4 + H3N5F1 + H4N5 + H4N4Gl1 + H5N4F1 + H4N4Ge1 + H4N5F1 + H4N4Ge1Ac1 + H4N4F1Gl1 + H5N4Gl1 + H4N4F1Ge1 + H5N4Ge1 + H5N4Ge1Ac1 + H4N5Ge1Ac1 + H4N4F2Gl1 + H5N4F1Gl1 + H5N4F1Ge1 + H5N4Gl2 + H5N4E1Gl1 + H5N4L1Ge1 + H5N4F2E1 + H5N4Ge1Gl1 + H6N5Gl1 + H5N4Ge1Gl1Ac1 + H5N4Ge2 + H5N4Ge2Ac1 + H4N4Ge1Gl2 + H5N4Ge2Ac2 + H5N4F1Ge1Gl1 + H5N4F1L1Ge1Ac1 + H5N4F1Ge2 + H5N4F1Ge2Ac1 + H5N4F1Ge2Ac2 + H5N4Ge1Gl2 + H5N4L1Ge2 + H5N4Ge2Gl1 + H6N5Ge1Gl1 + H5N4Ge2Gl1Ac1 + H5N4Ge2Gl1Ac2 + H5N4F1Ge1Gl2 + H5N4F1Ge2Gl1 + H6N5F1Ge1Gl1 + H5N4F1Ge2Gl1Ac1 + H5N4F1Ge2Gl1Ac2 + H5N4Ge2Gl2 + H6N5Ge1Gl2 + H6N5Ge1Gl2Ac1 + H6N5Ge2Gl1 + H5N4F1Ge1Gl3 + H6N5Ge1Gl2Ac2 + H6N5Ge2Gl1Ac1 + H6N5Ge3 + H5N4F1Ge2Gl2 + H6N5Ge1Gl2Ac3 + H6N5Ge2Gl1Ac2 + H6N5F1Ge1Gl2 + H6N5F1Ge1Gl2Ac1 + H6N5F1Ge2Gl1 + H6N5F1Ge1Gl2Ac2 + H6N5F1Ge1Gl2Ac3 + H6N5Ge1Gl3 + H6N5Ge2Gl2 + H7N6Ge1Gl2 + H6N5Ge2Gl2Ac1 + H6N5F1Ge1Gl3 + H6N5F1Ge2Gl2 + H7N6Ge1Gl3 / T |
| A1 | Monoantennary glycans | H3N3F1 + H3N3Ge1 + H3N3Ge1Ac1 + H4N3Gl1 + H4N3E1 + H4N3Ge1 + H4N3Ge1Ac1 + H5N3Gl1 + H3N3Ge1Gl1 + H4N3F1Ge1 + H5N3Ge1+ H6N3Gl1 + H4N3Ge1Gl1 + H6N3Ge1 + H5N3Ge1Gl1 + H5N3Ge2Ac1 / T |

| | | |
|---|---|---|
| A2 | Diantennary glycans | H3N4 + H3N4F1 + H4N4 + H4N4F1 + H5N4 + H3N5F1 + H4N5 + H4N4Gl1 + H5N4F1 + H4N4Ge1 + H4N5F1 + H4N4Ge1Ac1 + H4N4F1Gl1 + H5N4Gl1 + H4N4F1Ge1 + H5N4Ge1 + H5N4Ge1Ac1 + H4N5Ge1Ac1 + H4N4F2Gl1 + H5N4F1Gl1 + H5N4F1Ge1 + H5N4Gl2 + H5N4E1Gl1 + H5N4L1Ge1 + H5N4F2E1 + H5N4Ge1Gl1 + H5N4Ge1Gl1Ac1 + H5N4Ge2 + H5N4Ge2Ac1 + H4N4Ge1Gl2 + H5N4Ge2Ac2 + H5N4F1Ge1Gl1 + H5N4F1L1Ge1Ac1 + H5N4F1Ge2 + H5N4F1Ge2Ac1 + H5N4F1Ge2Ac2 + H5N4Ge1Gl2 + H5N4L1Ge2 + H5N4Ge2Gl1 + H5N4Ge2Gl1Ac1 + H5N4Ge2Gl1Ac2 + H5N4F1Ge1Gl2 + H5N4F1Ge2Gl1 + H5N4F1Ge2Gl1Ac1 + H5N4F1Ge2Gl1Ac2 + H5N4Ge2Gl2 + H5N4F1Ge1Gl3 + H5N4F1Ge2Gl2 / T |
| A3 | Triantennary glycans | H6N5Gl1 + H6N5Ge1Gl1 + H6N5F1Ge1Gl1 + H6N5Ge1Gl2 + H6N5Ge1Gl2Ac1 + H6N5Ge2Gl1 + H6N5Ge1Gl2Ac2 + H6N5Ge2Gl1Ac1 + H6N5Ge3 + H6N5Ge1Gl2Ac3 + H6N5Ge2Gl1Ac2 + H6N5F1Ge1Gl2 + H6N5F1Ge1Gl2Ac1 + H6N5F1Ge2Gl1 + H6N5F1Ge1Gl2Ac2 + H6N5F1Ge1Gl2Ac3 + H6N5Ge1Gl3 + H6N5Ge2Gl2 + H6N5Ge2Gl2Ac1 + H6N5F1Ge1Gl3 + H6N5F1Ge2Gl2 / T |
| A4 | Tetraantennary glycans | H7N6Ge1Gl2 + H7N6Ge1Gl3 / T |
| AG | Galactosylation per antenna across all glycan species | A2 * ((1/2 * (H4N4 + H4N4F1 + H4N5 + H4N4Gl1 + H4N4Ge1 + H4N5F1 + H4N4Ge1Ac1 + H4N4F1Gl1 + H4N4F1Ge1 + H4N5Ge1Ac1 + H4N4F2Gl1 + H4N4Ge1Gl2) + 2/2 * (H5N4 + H5N4F1 + H5N4Gl1 + H5N4Ge1 + H5N4Ge1Ac1 + H5N4F1Gl1 + H5N4F1Ge1 + H5N4Gl2 + H5N4E1Gl1 + H5N4L1Ge1 + H5N4F2E1 + H5N4Ge1Gl1 + H5N4Ge1Gl1Ac1 + H5N4Ge2 + H5N4Ge2Ac1 + H5N4Ge2Ac2 + H5N4F1Ge1Gl1 + H5N4F1L1Ge1Ac1 + H5N4F1Ge2 + H5N4F1Ge2Ac1 + H5N4F1Ge2Ac2 + H5N4Ge1Gl2 + H5N4L1Ge2 + H5N4Ge2Gl1 + H5N4Ge2Gl1Ac1 + H5N4Ge2Gl1Ac2 + H5N4F1Ge1Gl2 + H5N4F1Ge2Gl1 + H5N4F1Ge2Gl1Ac1 + H5N4F1Ge2Gl1Ac2 + H5N4Ge2Gl2 + H5N4F1Ge1Gl3 + H5N4F1Ge2Gl2)) / (1 * (H3N4 + H3N4F1 + H4N4 + H4N4F1 + H5N4 + H3N5F1 + H4N5 + H4N4Gl1 + H5N4F1 + H4N4Ge1 + H4N5F1 + H4N4Ge1Ac1 + H4N4F1Gl1 + H5N4Gl1 + H4N4F1Ge1 + H5N4Ge1 + H5N4Ge1Ac1 + H4N5Ge1Ac1 + H4N4F2Gl1 + H5N4F1Gl1 + H5N4F1Ge1 + H5N4Gl2 + H5N4E1Gl1 + H5N4L1Ge1 + H5N4F2E1 + H5N4Ge1Gl1 + H5N4Ge1Gl1Ac1 + H5N4Ge2 + H5N4Ge2Ac1 + H4N4Ge1Gl2 + H5N4Ge2Ac2 + H5N4F1Ge1Gl1 + H5N4F1L1Ge1Ac1 + H5N4F1Ge2 + H5N4F1Ge2Ac1 + H5N4F1Ge2Ac2 + H5N4Ge1Gl2 + H5N4L1Ge2 + H5N4Ge2Gl1 + H5N4Ge2Gl1Ac1 + H5N4Ge2Gl1Ac2 + H5N4F1Ge1Gl2 + H5N4F1Ge2Gl1 + H5N4F1Ge2Gl1Ac1 + H5N4F1Ge2Gl1Ac2 + H5N4Ge2Gl2 + H5N4F1Ge1Gl3 + H5N4F1Ge2Gl2))) + A3 * ((1/3 * (0) + 2/3 * (0) + 3/3 * (H6N5Gl1 + H6N5Ge1Gl1 + H6N5F1Ge1Gl1 + H6N5Ge1Gl2 + H6N5Ge1Gl2Ac1 + H6N5Ge2Gl1 + H6N5Ge1Gl2Ac2 + H6N5Ge2Gl1Ac1 + H6N5Ge3 + H6N5Ge1Gl2Ac3 + H6N5Ge2Gl1Ac2 + H6N5F1Ge1Gl2 + H6N5F1Ge1Gl2Ac1 + H6N5F1Ge2Gl1 + H6N5F1Ge1Gl2Ac2 + H6N5F1Ge1Gl2Ac3 + H6N5Ge1Gl3 + H6N5Ge2Gl2 + H6N5Ge2Gl2Ac1 + H6N5F1Ge1Gl3 + H6N5F1Ge2Gl2)) / (1 * (H6N5Gl1 + H6N5Ge1Gl1 + H6N5F1Ge1Gl1 + H6N5Ge1Gl2 + H6N5Ge1Gl2Ac1 + H6N5Ge2Gl1 + H6N5Ge1Gl2Ac2 + H6N5Ge2Gl1Ac1 + H6N5Ge3 + H6N5Ge1Gl2Ac3 + H6N5Ge2Gl1Ac2 + H6N5F1Ge1Gl2 + H6N5F1Ge1Gl2Ac1 + H6N5F1Ge2Gl1 + H6N5F1Ge1Gl2Ac2 + H6N5F1Ge1Gl2Ac3 + H6N5Ge1Gl3 + H6N5Ge2Gl2 + H6N5Ge2Gl2Ac1 + H6N5F1Ge1Gl3 + H6N5F1Ge2Gl2)) + A4 * ((4/4 * (H7N6Ge1Gl2 + H7N6Ge1Gl3)) / (1 * (H7N6Ge1Gl2 + H7N6Ge1Gl3))) / (A2 + A3 + A4) |
| F | Fucosylated glycans | H3N3F1 + H4N3F1 + H3N4F1 + H4N4F1 + H3N5F1 + H4N3F1Ge1 + H5N4F1 + H4N5F1 + H4N4F1Gl1 + H4N4F1Ge1 + H4N4F2Gl1 + H5N4F1Gl1 + H5N4F1Ge1 + H5N4F2E1 + H5N4F1Ge1Gl1 + H5N4F1L1Ge1Ac1 + H5N4F1Ge2 + H5N4F1Ge2Ac1 + H5N4F1Ge2Ac2 + H5N4F1Ge1Gl2 + H5N4F1Ge2Gl1 + H6N5F1Ge1Gl1 + H5N4F1Ge2Gl1Ac1 + H5N4F1Ge2Gl1Ac2 + H5N4F1Ge1Gl3 + H5N4F1Ge2Gl2 + H6N5F1Ge1Gl2 + H6N5F1Ge1Gl2Ac1 + H6N5F1Ge2Gl1 + H6N5F1Ge1Gl2Ac2 + H6N5F1Ge1Gl2Ac3 + H6N5F1Ge1Gl3 + H6N5F1Ge2Gl2 / T |
| Fa | Difucosylated glycans | H4N4F2Gl1 + H5N4F2E1 / T |
| TS | sialic acids (NeuAc & NeuGc) per antenna across all glycan species | ANGnaS + AS |

| | | |
|---|---|---|
| ANGnaS | NeuGc per antenna across all glycan species | ANGnaL+ ANGnaE |
| ANGnaL | α-2,3-linked NeuGc per antenna across all glycan species | (A2 * ((1/2 * (H4N4Gl1 + H4N4F1Gl1 + H5N4Gl1 + H4N4F2Gl1 + H5N4F1Gl1 + H5N4E1Gl1 + H5N4Ge1Gl1 + H5N4Ge1Gl1Ac1 + H5N4F1Ge1Gl1 + H5N4Ge2Gl1 + H5N4Ge2Gl1Ac1 + H5N4Ge2Gl1Ac2 + H5N4F1Ge2Gl1 + H5N4F1Ge2Gl1Ac1 + H5N4F1Ge2Gl1Ac2) + 2/2 * (H5N4Gl2 + H4N4Ge1Gl2 + H5N4Ge1Gl2 + H5N4F1Ge1Gl2 + H5N4Ge2Gl2 + H5N4F1Ge2Gl2) + 3/2 * (H5N4F1Ge1Gl3) + 4/2 * (0)) / (1 * (H3N4 + H3N4F1 + H4N4 + H4N4F1 + H5N4 + H3N5F1 + H4N5 + H4N4Gl1 + H5N4F1 + H4N4Ge1 + H4N5F1 + H4N4Ge1Ac1 + H4N4F1Gl1 + H5N4Gl1 + H4N4F1Ge1 + H5N4Ge1 + H5N4Ge1Ac1 + H4N5Ge1Ac1 + H4N4F2Gl1 + H5N4F1Gl1 + H5N4F1Ge1 + H5N4Gl2 + H5N4E1Gl1 + H5N4L1Ge1 + H5N4F2E1 + H5N4Ge1Gl1 + H5N4Ge1Gl1Ac1 + H5N4Ge2 + H5N4Ge2Ac1 + H4N4Ge1Gl2 + H5N4Ge2Ac2 + H5N4F1Ge1Gl1 + H5N4F1L1Ge1Ac1 + H5N4F1Ge2 + H5N4F1Ge2Ac1 + H5N4F1Ge2Ac2 + H5N4Ge1Gl2 + H5N4L1Ge2 + H5N4Ge2Gl1 + H5N4Ge2Gl1Ac1 + H5N4Ge2Gl1Ac2 + H5N4F1Ge1Gl2 + H5N4F1Ge2Gl1 + H5N4F1Ge2Gl1Ac1 + H5N4F1Ge2Gl1Ac2 + H5N4Ge2Gl2 + H5N4F1Ge1Gl3 + H5N4F1Ge2Gl2)).) + A3 * ((1/3 * (H6N5Gl1 + H6N5Ge1Gl1 + H6N5F1Ge1Gl1 + H6N5Ge2Gl1 + H6N5Ge2Gl1Ac1 + H6N5Ge2Gl1Ac2 + H6N5F1Ge2Gl1) + 2/3 * (H6N5Ge1Gl2 + H6N5Ge1Gl2Ac1 + H6N5Ge1Gl2Ac2 + H6N5Ge1Gl2Ac3 + H6N5F1Ge1Gl2 + H6N5F1Ge1Gl2Ac1 + H6N5F1Ge1Gl2Ac2 + H6N5F1Ge1Gl2Ac3 + H6N5Ge2Gl2 + H6N5Ge2Gl2Ac1 + H6N5F1Ge2Gl2) + 3/3 * (H6N5Ge1Gl3 + H6N5F1Ge1Gl3) + 4/3 * (0) + 5/3 * (0)) / (1 * (H6N5Gl1 + H6N5Ge1Gl1 + H6N5F1Ge1Gl1 + H6N5Ge1Gl2 + H6N5Ge1Gl2Ac1 + H6N5Ge2Gl1 + H6N5Ge1Gl2Ac2 + H6N5Ge2Gl1Ac1 + H6N5Ge3 + H6N5Ge1Gl2Ac3 + H6N5Ge2Gl1Ac2 + H6N5F1Ge1Gl2 + H6N5F1Ge1Gl2Ac1 + H6N5F1Ge2Gl1 + H6N5F1Ge1Gl2Ac2 + H6N5F1Ge1Gl2Ac3 + H6N5Ge1Gl3 + H6N5Ge2Gl2 + H6N5Ge2Gl2Ac1 + H6N5F1Ge1Gl3 + H6N5F1Ge2Gl2))) + A4 * ((1/4 * (0) + 2/4 * (H7N6Ge1Gl2) + 3/4 * H7N6Ge1Gl3) + 4/4 * (0) + 5/4 * (0) + 6/4 * (0)) / (1 * (H7N6Ge1Gl2 + H7N6Ge1Gl3)))) / (1 * (A2 + A3 + A4)). |
| ANGnaE | α-2,6-linked NeuGc per antenna across all glycan species | (A2 * ((1/2 * (H4N4Ge1 + H4N4Ge1Ac1 + H4N4F1Ge1 + H5N4Ge1 + H5N4Ge1Ac1 + H4N5Ge1Ac1 + H5N4F1Ge1 + H5N4L1Ge1 + H5N4Ge1Gl1 + H5N4Ge1Gl1Ac1 + H4N4Ge1Gl2 + H5N4F1Ge1Gl1 + H5N4F1L1Ge1Ac1 + H5N4Ge1Gl2 + H5N4F1Ge1Gl2 + H5N4F1Ge1Gl3) + 2/2 * (H5N4Ge2 + H5N4Ge2Ac1 + H5N4Ge2Ac2 + H5N4F1Ge2 + H5N4F1Ge2Ac1 + H5N4F1Ge2Ac2 + H5N4L1Ge2 + H5N4Ge2Gl1 + H5N4Ge2Gl1Ac1 + H5N4Ge2Gl1Ac2 + H5N4F1Ge2Gl1 + H5N4F1Ge2Gl1Ac1 + H5N4F1Ge2Gl1Ac2 + H5N4Ge2Gl2 + H5N4F1Ge2Gl2) + 3/2 * (0) + 4/2 * (0)) / (1 * (H3N4 + H3N4F1 + H4N4 + H4N4F1 + H5N4 + H3N5F1 + H4N5 + H4N4Gl1 + H5N4F1 + H4N4Ge1 + H4N5F1 + H4N4Ge1Ac1 + H4N4F1Gl1 + H5N4Gl1 + H4N4F1Ge1 + H5N4Ge1 + H5N4Ge1Ac1 + H4N5Ge1Ac1 + H4N4F2Gl1 + H5N4F1Gl1 + H5N4F1Ge1 + H5N4Gl2 + H5N4E1Gl1 + H5N4L1Ge1 + H5N4F2E1 + H5N4Ge1Gl1 + H5N4Ge1Gl1Ac1 + H5N4Ge2 + H5N4Ge2Ac1 + H4N4Ge1Gl2 + H5N4Ge2Ac2 + H5N4F1Ge1Gl1 + H5N4F1L1Ge1Ac1 + H5N4F1Ge2 + H5N4F1Ge2Ac1 + H5N4F1Ge2Ac2 + H5N4Ge1Gl2 + H5N4L1Ge2 + H5N4Ge2Gl1 + H5N4Ge2Gl1Ac1 + H5N4Ge2Gl1Ac2 + 5N4F1Ge1Gl2 + H5N4F1Ge2Gl1 + H5N4F1Ge2Gl1Ac1 + H5N4F1Ge2Gl1Ac2 + H5N4Ge2Gl2 + H5N4F1Ge1Gl3 + H5N4F1Ge2Gl2))) + A3 * ((1/3 * (H6N5Ge1Gl1 + H6N5F1Ge1Gl1 + H6N5Ge1Gl2 + H6N5Ge1Gl2Ac1 + H6N5Ge1Gl2Ac2 + H6N5Ge1Gl2Ac3 + H6N5F1Ge1Gl2 + H6N5F1Ge1Gl2Ac1 + H6N5F1Ge1Gl2Ac2 + H6N5F1Ge1Gl2Ac3 + H6N5Ge1Gl3 + H6N5F1Ge1Gl3) + 2/3 * (H6N5Ge2Gl1 + H6N5Ge2Gl1Ac1 + H6N5Ge2Gl1Ac2 + H6N5F1Ge2Gl1 + H6N5Ge2Gl2 + H6N5Ge2Gl2Ac1 + H6N5F1Ge2Gl2) + 3/3 * (H6N5Ge3) + 4/3 * (0) + 5/3 * (0)) / (1 * (H6N5Gl1 + H6N5Ge1Gl1 + H6N5F1Ge1Gl1 + H6N5Ge1Gl2 + H6N5Ge1Gl2Ac1 + H6N5Ge2Gl1 + H6N5Ge1Gl2Ac2 + H6N5Ge2Gl1Ac1 + H6N5Ge3 + H6N5Ge1Gl2Ac3 + H6N5Ge2Gl1Ac2 + H6N5F1Ge1Gl2 + H6N5F1Ge1Gl2Ac1 + H6N5F1Ge2Gl1 + H6N5F1Ge1Gl2Ac2 + H6N5F1Ge1Gl2Ac3 + H6N5Ge1Gl3 + H6N5Ge2Gl2 + H6N5Ge2Gl2Ac1 + H6N5F1Ge1Gl3 + H6N5F1Ge2Gl2))) + A4 * ((1/4 * (H7N6Ge1Gl2 + H7N6Ge1Gl3) + 2/4 * (0) + 3/4 * (0) + 4/4 * (0) + 5/4 * (0) + 6/4 * (0)) / (1 * (H7N6Ge1Gl2 + H7N6Ge1Gl3))) / (1 * (A2 + A3 + A4)). |
| AS | NeuAc per antenna across all glycan species | AL+AE |

| | | |
|---|---|---|
| AL | α-2,3--linked NeuAc per antenna across all glycan species | (A2 * ((1/2 * (H5N4L1Ge1 + H5N4F1L1Ge1Ac1 + H5N4L1Ge2) + 2/2 * (0) + 3/2 * (0) + 4/2 * (0)) / (1 * (H3N4 + H3N4F1 + H4N4 + H4N4F1 + H5N4 + H3N5F1 + H4N5 + H4N4Gl1 + H5N4F1 + H4N4Ge1 + H4N5F1 + H4N4Ge1Ac1 + H4N4F1Gl1 + H5N4Gl1 + H4N4F1Ge1 + H5N4Ge1 + H5N4Ge1Ac1 + H4N5Ge1Ac1 + H4N4F2Gl1 + H5N4F1Gl1 + H5N4F1Ge1 + H5N4Gl2 + H5N4E1Gl1 + H5N4L1Ge1 + H5N4F2E1 + H5N4Ge1Gl1 + H5N4Ge1Gl1Ac1 + H5N4Ge2 + H5N4Ge2Ac1 + H4N4Ge1Gl2 + H5N4Ge2Ac2 + H5N4F1Ge1Gl1 + H5N4F1L1Ge1Ac1 + H5N4F1Ge2 + H5N4F1Ge2Ac1 + H5N4F1Ge2Ac2 + H5N4Ge1Gl2 + H5N4L1Ge2 + H5N4Ge2Gl1 + H5N4Ge2Gl1Ac1 + H5N4Ge2Gl1Ac2 + H5N4F1Ge1Gl2 + H5N4F1Ge2Gl1 + H5N4F1Ge2Gl1Ac1 + H5N4F1Ge2Gl1Ac2 + H5N4Ge2Gl2 + H5N4F1Ge1Gl3 + H5N4F1Ge2Gl2)).) + A3 * ((1/3 * (0) + 2/3 * (0) + 3/3 * (0) + 4/3 * (0) + 5/3 * (0)) / (1 * (H6N5Gl1 + H6N5Ge1Gl1 + H6N5F1Ge1Gl1 + H6N5Ge1Gl2 + H6N5Ge1Gl2Ac1 + H6N5Ge2Gl1 + H6N5Ge1Gl2Ac2 + H6N5Ge2Gl1Ac1 + H6N5Ge3 + H6N5Ge1Gl2Ac3 + H6N5Ge2Gl1Ac2 + H6N5F1Ge1Gl2 + H6N5F1Ge1Gl2Ac1 + H6N5F1Ge2Gl1 + H6N5F1Ge1Gl2Ac2 + H6N5F1Ge1Gl2Ac3 + H6N5Ge1Gl3 + H6N5Ge2Gl2 + H6N5Ge2Gl2Ac1 + H6N5F1Ge1Gl3 + H6N5F1Ge2Gl2))) + A4 * ((1/4 * (0) + 2/4 * (0) + 3/4 * (0) + 4/4 * (0) + 5/4 * (0) + 6/4 * (0)) / (1 * (H7N6Ge1Gl2 + H7N6Ge1Gl3)))) / (1 * (A2 + A3 + A4)). |
| AE | α-2,6-linked NeuAc per antenna across all glycan species | (A2 * ((1/2 * (H5N4E1Gl1 + H5N4F2E1) + 2/2 * (0) + 3/2 * (0) + 4/2 * (0)) / (1 * (H3N4 + H3N4F1 + H4N4 + H4N4F1 + H5N4 + H3N5F1 + H4N5 + H4N4Gl1 + H5N4F1 + H4N4Ge1 + H4N5F1 + H4N4Ge1Ac1 + H4N4F1Gl1 + H5N4Gl1 + H4N4F1Ge1 + H5N4Ge1 + H5N4Ge1Ac1 + H4N5Ge1Ac1 + H4N4F2Gl1 + H5N4F1Gl1 + H5N4F1Ge1 + H5N4Gl2 + H5N4E1Gl1 + H5N4L1Ge1 + H5N4F2E1 + H5N4Ge1Gl1 + H5N4Ge1Gl1Ac1 + H5N4Ge2 + H5N4Ge2Ac1 + H4N4Ge1Gl2 + H5N4Ge2Ac2 + H5N4F1Ge1Gl1 + H5N4F1L1Ge1Ac1 + H5N4F1Ge2 + H5N4F1Ge2Ac1 + H5N4F1Ge2Ac2 + H5N4Ge1Gl2 + H5N4L1Ge2 + H5N4Ge2Gl1 + H5N4Ge2Gl1Ac1 + H5N4Ge2Gl1Ac2 + H5N4F1Ge1Gl2 + H5N4F1Ge2Gl1 + H5N4F1Ge2Gl1Ac1 + H5N4F1Ge2Gl1Ac2 + H5N4Ge2Gl2 + H5N4F1Ge1Gl3 + H5N4F1Ge2Gl2))) + A3 * ((1/3 * (0) + 2/3 * (0) + 3/3 * (0) + 4/3 * (0) + 5/3 * (0)) / (1 * (H6N5Gl1 + H6N5Ge1Gl1 + H6N5F1Ge1Gl1 + H6N5Ge1Gl2 + H6N5Ge1Gl2Ac1 + H6N5Ge2Gl1 + H6N5Ge1Gl2Ac2 + H6N5Ge2Gl1Ac1 + H6N5Ge3 + H6N5Ge1Gl2Ac3 + H6N5Ge2Gl1Ac2 + H6N5F1Ge1Gl2 + H6N5F1Ge1Gl2Ac1 + H6N5F1Ge2Gl1 + H6N5F1Ge1Gl2Ac2 + H6N5F1Ge1Gl2Ac3 + H6N5Ge1Gl3 + H6N5Ge2Gl2 + H6N5Ge2Gl2Ac1 + H6N5F1Ge1Gl3 + H6N5F1Ge2Gl2))) + A4 * ((1/4 * (0) + 2/4 * (0) + 3/4 * (0) + 4/4 * (0) + 5/4 * (0) + 6/4 * (0)) / (1 * (H7N6Ge1Gl2 + H7N6Ge1Gl3)))) / (1 * (A2 + A3 + A4)). |
| TsAc | Acetylation of sialic acids (NeuAc & NeuGc) | 1/1 * (H3N3Ge1Ac1 + H4N3Ge1Ac1 + H4N4Ge1Ac1 + H5N4Ge1Ac1 + H4N5Ge1Ac1) + 1/2 * (H5N3Ge2Ac1 + H5N4Ge1Gl1Ac1 + H5N4Ge2Ac1 + H5N4F1L1Ge1Ac1 + H5N4F1Ge2Ac1) + 1/3 * (H5N4Ge2Gl1Ac1 + H5N4F1Ge2Gl1Ac1 + H6N5Ge1Gl2Ac1 + H6N5Ge2Gl1Ac1 + H6N5F1Ge1Gl2Ac1) + 1/4 * (H6N5Ge2Gl2Ac1) + 2/2 * (H5N4Ge2Ac2 + H5N4F1Ge2Ac2) + 2/3 * (H5N4Ge2Gl1Ac2 + H5N4F1Ge2Gl1Ac2 + H6N5Ge1Gl2Ac2 + H6N5Ge2Gl1Ac2 + H6N5F1Ge1Gl2Ac2) 3/3 * (H6N5Ge1Gl2Ac3 + H6N5F1Ge1Gl2Ac3) / T |
| Tsb | Branching sialic acids (NeuGc) across all glycan species | (A1 * ((H3N3Ge1Gl1 + H4N3Ge1Gl1 + H5N3Ge1Gl1 + H5N3Ge2Ac1)) / ((H3N3F1 + H3N3Ge1 + H3N3Ge1Ac1 + H4N3Gl1 + H4N3E1 + H4N3Ge1 + H4N3Ge1Ac1 + H3N3Ge1Gl1 + H4N3F1Ge1 + H4N3Ge1Gl1 + H5N3Ge1Gl1 + H5N3Ge2Ac1)) + A2 * ((1 * (H4N4Ge1Gl2 + H5N4Ge1Gl2 + H5N4Ge2Gl1 + H5N4Ge2Gl1Ac1 + H5N4Ge2Gl1Ac2 + H5N4F1Ge1Gl2 + H5N4F1Ge2Gl1 + H5N4F1Ge2Gl1Ac1 + H5N4F1Ge2Gl1Ac2 + H5N4Ge2Gl2 + H5N4F1Ge1Gl3 + 5N4F1Ge2Gl2)) / (1 * (H3N4 + H3N4F1 + H4N4 + H4N4F1 + H5N4 + H3N5F1 + H4N5 + H4N4Gl1 + H5N4F1 + H4N4Ge1 + H4N5F1 + H4N4Ge1Ac1 + H4N4F1Gl1 + H5N4Gl1 + H4N4F1Ge1 + H5N4Ge1 + H5N4Ge1Ac1 + H4N5Ge1Ac1 + H4N4F2Gl1 + H5N4F1Gl1 + H5N4F1Ge1 + H5N4Gl2 + H5N4E1Gl1 + H5N4L1Ge1 + H5N4F2E1 + H5N4Ge1Gl1 + H5N4Ge1Gl1Ac1 + H5N4Ge2 + H5N4Ge2Ac1 + H4N4Ge1Gl2 + H5N4Ge2Ac2 + H5N4F1Ge1Gl1 + H5N4F1L1Ge1Ac1 + H5N4F1Ge2 + H5N4F1Ge2Ac1 + H5N4F1Ge2Ac2 + H5N4Ge1Gl2 + H5N4L1Ge2 + H5N4Ge2Gl1 + H5N4Ge2Gl1Ac1 + H5N4Ge2Gl1Ac2 + H5N4F1Ge1Gl2 + H5N4F1Ge2Gl1 + H5N4F1Ge2Gl1Ac1 + H5N4F1Ge2Gl1Ac2 + H5N4Ge2Gl2 + H5N4F1Ge1Gl3 + H5N4F1Ge2Gl2))) + A3 * ((1 * (H6N5Ge1Gl3 + H6N5Ge2Gl2 + H6N5Ge2Gl2Ac1 + H6N5F1Ge1Gl3 + H6N5F1Ge2Gl2)) / (1 * (H6N5Gl1 + H6N5Ge1Gl1 + H6N5F1Ge1Gl1 + H6N5Ge1Gl2 + H6N5Ge1Gl2Ac1 + H6N5Ge2Gl1 + H6N5Ge1Gl2Ac2 + H6N5Ge2Gl1Ac1 + H6N5Ge3 + H6N5Ge1Gl2Ac3 + H6N5Ge2Gl1Ac2 + H6N5F1Ge1Gl2 + H6N5F1Ge1Gl2Ac1 + H6N5F1Ge2Gl1 + H6N5F1Ge1Gl2Ac2 + H6N5F1Ge1Gl2Ac3 + H6N5Ge1Gl3 + H6N5Ge2Gl2 + H6N5Ge2Gl2Ac1 + H6N5F1Ge1Gl3 + H6N5F1Ge2Gl2))) + A4 * ((1 * (0)) / (1 * (H7N6Ge1Gl2 + H7N6Ge1Gl3)))) / (1 * (A1 + A2 + A3 + A4)) |

Supplementary Table S2: Overview of the 94 *N*-glycan monosaccharide compositions and substituents identified and their relative abundances in TPNG and TPFG of mice (n=3 per group; total 6) at the starting point (0h) of the experiment 1. H = hexose; N = *N*-acetylhexosamine; F = fucose; E = α2,6-linked *N*-acetylneuraminic acid; L= α2,3-linked *N*-acetylneuraminic acid; Ge = α2,6-linked *N*-glycolylneuraminic acid; Gl= α2,3-linked *N*-glycolylneuraminic acid; Ac = acetyl group.

| | | Plasma (TPNG) | | Peritoneal Fluid (TPFG) | |
|---|---|---|---|---|---|
| | | Relative abundance (%) | | Relative abundance (%) | |
| Glycan compositions | [M+Na]+ | Mean | SEM | Mean | SEM |
| H5N2 | 1257.42 | 0.12 | 0.0 | 0.20 | 0.0 |
| H3N3F1 | 1282.45 | 0.02 | 0.0 | 0.10 | 0.0 |
| H4N3 | 1298.45 | 0.03 | 0.0 | 0.11 | 0.0 |
| H3N4 | 1339.48 | 0.02 | 0.0 | 0.20 | 0.0 |
| H6N2 | 1419.48 | 0.27 | 0.0 | 0.54 | 0.1 |
| H4N3F1 | 1444.51 | 0.02 | 0.0 | 0.14 | 0.0 |
| H3N3Ge1 | 1471.52 | 0.16 | 0.0 | 0.20 | 0.0 |
| H3N4F1 | 1485.53 | 0.19 | 0.1 | 0.29 | 0.0 |
| H4N4 | 1501.53 | 0.08 | 0.0 | 0.27 | 0.1 |
| H3N3Ge1Ac1 | 1513.53 | 0.02 | 0.0 | 0.21 | 0.0 |
| H7N2 | 1581.53 | 0.09 | 0.0 | 0.16 | 0.0 |
| H4N3Gl1 | 1587.53 | 0.07 | 0.0 | 0.31 | 0.0 |
| H4N3E1 | 1617.58 | 0.12 | 0.0 | 0.38 | 0.1 |
| H4N3Ge1 | 1633.57 | 0.36 | 0.0 | 0.54 | 0.1 |
| H4N4F1 | 1647.59 | 0.35 | 0.1 | 0.46 | 0.1 |
| H5N4 | 1663.58 | 0.09 | 0.0 | 0.17 | 0.0 |
| H4N3Ge1Ac1 | 1675.58 | 0.04 | 0.0 | 0.11 | 0.0 |
| H3N5F1 | 1688.61 | 0.04 | 0.0 | 0.17 | 0.0 |
| H4N5 | 1704.61 | 0.04 | 0.0 | 0.17 | 0.0 |
| H8N2 | 1743.58 | 0.12 | 0.0 | 0.22 | 0.0 |
| H5N3Gl1 | 1749.58 | 0.14 | 0.0 | 0.18 | 0.1 |
| H3N3Ge1Gl1 | 1760.60 | 0.03 | 0.0 | 0.08 | 0.0 |
| H4N3F1Ge1 | 1779.63 | 0.20 | 0.0 | 0.30 | 0.1 |
| H4N4Gl1 | 1790.61 | 0.16 | 0.0 | 0.17 | 0.0 |
| H5N3Ge1 | 1795.62 | 0.38 | 0.0 | 0.34 | 0.0 |
| H5N4F1 | 1809.64 | 0.18 | 0.0 | 0.36 | 0.1 |
| H4N4Ge1 | 1836.65 | 0.74 | 0.0 | 0.57 | 0.1 |
| H4N5F1 | 1850.67 | 0.05 | 0.0 | 0.12 | 0.1 |
| H4N4Ge1Ac1 | 1878.66 | 0.04 | 0.0 | 0.09 | 0.0 |
| H9N2 | 1905.63 | 0.33 | 0.1 | 0.32 | 0.0 |
| H6N3Gl1 | 1911.63 | 0.20 | 0.0 | 0.20 | 0.0 |
| H4N3Ge1Gl1 | 1922.65 | 0.15 | 0.0 | 0.24 | 0.0 |

| | | | | | |
|---|---|---|---|---|---|
| H4N4F1Gl1 | 1936.67 | 0.07 | 0.0 | 0.14 | 0.1 |
| H5N4Gl1 | 1952.66 | 0.45 | 0.1 | 0.40 | 0.0 |
| H6N3Ge1 | 1957.68 | 0.37 | 0.0 | 0.22 | 0.0 |
| H4N4F1Ge1 | 1982.71 | 0.26 | 0.0 | 0.33 | 0.1 |
| H5N4Ge1 | 1998.70 | 3.34 | 0.7 | 1.40 | 0.1 |
| H5N4Ge1Ac1 | 2040.71 | 0.21 | 0.0 | 0.21 | 0.1 |
| H4N5Ge1Ac1 | 2081.74 | 0.11 | 0.0 | 0.19 | 0.0 |
| H4N4F2Gl1 | 2082.72 | 0.13 | 0.0 | 0.18 | 0.0 |
| H5N3Ge1Gl1 | 2084.70 | 0.06 | 0.0 | 0.12 | 0.0 |
| H5N4F1Gl1 | 2098.72 | 0.25 | 0.0 | 0.25 | 0.0 |
| H5N4F1Ge1 | 2144.76 | 1.42 | 0.2 | 0.82 | 0.2 |
| H5N3Ge2Ac1 | 2172.76 | 0.28 | 0.0 | 0.22 | 0.0 |
| H5N4Gl2 | 2241.74 | 0.59 | 0.0 | 0.56 | 0.0 |
| H5N4E1Gl1 | 2271.79 | 0.24 | 0.0 | 0.18 | 0.0 |
| H5N4L1Ge1 | 2271.79 | 0.24 | 0.0 | 0.18 | 0.0 |
| H5N4F2E1 | 2274.82 | 0.09 | 0.0 | 0.10 | 0.0 |
| H5N4Ge1Gl1 | 2287.78 | 10.30 | 0.3 | 9.44 | 0.3 |
| H6N5Gl1 | 2317.79 | 0.50 | 0.0 | 0.39 | 0.1 |
| H5N4Ge1Gl1Ac1 | 2329.79 | 1.80 | 0.1 | 1.98 | 0.0 |
| H5N4Ge2 | 2333.82 | 31.77 | 1.0 | 30.37 | 2.0 |
| H5N4Ge2Ac1 | 2375.84 | 3.66 | 0.3 | 4.72 | 0.5 |
| H4N4Ge1Gl2 | 2414.81 | 0.15 | 0.0 | 0.14 | 0.0 |
| H5N4Ge2Ac2 | 2417.85 | 1.12 | 0.1 | 1.44 | 0.1 |
| H5N4F1Ge1Gl1 | 2433.84 | 4.83 | 0.3 | 4.70 | 0.6 |
| H5N4F1L1Ge1Ac1 | 2459.86 | 0.29 | 0.0 | 0.32 | 0.0 |
| H5N4F1Ge2 | 2479.88 | 15.89 | 1.2 | 14.36 | 1.7 |
| H5N4F1Ge2Ac1 | 2521.89 | 2.53 | 0.3 | 2.97 | 0.2 |
| H5N4F1Ge2Ac2 | 2563.90 | 0.70 | 0.1 | 0.99 | 0.1 |
| H5N4Ge1Gl2 | 2576.86 | 0.43 | 0.0 | 0.59 | 0.0 |
| H5N4L1Ge2 | 2606.91 | 0.27 | 0.0 | 0.25 | 0.0 |
| H5N4Ge2Gl1 | 2622.90 | 1.92 | 0.1 | 2.17 | 0.1 |
| H6N5Ge1Gl1 | 2652.91 | 0.91 | 0.3 | 0.24 | 0.1 |
| H5N4Ge2Gl1Ac1 | 2664.91 | 0.39 | 0.0 | 0.58 | 0.0 |
| H5N4Ge2Gl1Ac2 | 2706.93 | 0.15 | 0.0 | 0.28 | 0.0 |
| H5N4F1Ge1Gl2 | 2722.92 | 0.27 | 0.0 | 0.36 | 0.1 |
| H5N4F1Ge2Gl1 | 2768.96 | 0.84 | 0.1 | 0.95 | 0.1 |
| H6N5F1Ge1Gl1 | 2798.97 | 0.33 | 0.1 | 0.07 | 0.0 |
| H5N4F1Ge2Gl1Ac1 | 2810.97 | 0.20 | 0.0 | 0.29 | 0.0 |
| H5N4F1Ge2Gl1Ac2 | 2852.98 | 0.13 | 0.0 | 0.28 | 0.1 |
| H5N4Ge2Gl2 | 2911.98 | 0.09 | 0.0 | 0.15 | 0.0 |
| H6N5Ge1Gl2 | 2941.99 | 2.48 | 0.1 | 2.11 | 0.4 |
| H6N5Ge1Gl2Ac1 | 2984.01 | 0.54 | 0.0 | 0.66 | 0.1 |
| H6N5Ge2Gl1 | 2988.04 | 1.41 | 0.0 | 1.32 | 0.1 |

| | | | | | |
|---|---|---|---|---|---|
| H5N4F1Ge1Gl3 | 3012.00 | 0.07 | 0.0 | 0.11 | 0.0 |
| H6N5Ge1Gl2Ac2 | 3026.02 | 0.19 | 0.0 | 0.37 | 0.0 |
| H6N5Ge2Gl1Ac1 | 3030.05 | 0.25 | 0.0 | 0.44 | 0.0 |
| H6N5Ge3 | 3034.08 | 0.15 | 0.0 | 0.24 | 0.0 |
| H5N4F1Ge2Gl2 | 3058.04 | 0.06 | 0.0 | 0.13 | 0.0 |
| H6N5Ge1Gl2Ac3 | 3068.03 | 0.07 | 0.0 | 0.13 | 0.0 |
| H6N5Ge2Gl1Ac2 | 3072.06 | 0.13 | 0.0 | 0.21 | 0.0 |
| H6N5F1Ge1Gl2 | 3088.05 | 0.83 | 0.1 | 0.73 | 0.2 |
| H6N5F1Ge1Gl2Ac1 | 3130.06 | 0.24 | 0.0 | 0.35 | 0.0 |
| H6N5F1Ge2Gl1 | 3134.09 | 0.40 | 0.0 | 0.39 | 0.1 |
| H6N5F1Ge1Gl2Ac2 | 3172.07 | 0.11 | 0.0 | 0.22 | 0.0 |
| H6N5F1Ge1Gl2Ac3 | 3214.08 | 0.05 | 0.0 | 0.08 | 0.0 |
| H6N5Ge1Gl3 | 3231.07 | 0.12 | 0.0 | 0.24 | 0.0 |
| H6N5Ge2Gl2 | 3277.12 | 0.19 | 0.0 | 0.23 | 0.0 |
| H7N6Ge1Gl2 | 3307.13 | 0.03 | 0.0 | 0.07 | 0.0 |
| H6N5Ge2Gl2Ac1 | 3319.13 | 0.07 | 0.0 | 0.15 | 0.0 |
| H6N5F1Ge1Gl3 | 3377.13 | 0.04 | 0.0 | 0.12 | 0.0 |
| H6N5F1Ge2Gl2 | 3423.17 | 0.06 | 0.0 | 0.10 | 0.0 |
| H7N6Ge1Gl3 | 3596.21 | 0.06 | 0.0 | 0.10 | 0.0 |

Supplementary Table S3: Overview of the 20 derived *N*-glycosylation traits and their relative abundances in total plasma *N*-glycome (TPNG) and total peritoneal fluid *N*-glycome (TPFG) of mice (n = 3 per group; total 6) at the starting point (0h) of the experiment 1. Paired t-tes was used to compare TPNG and TPFG glycosylation traits. NeuAc= *N*-acetylneuraminic acid; NeuGc= *N*-glycolylneuraminic acid; NS= not significant.

| | TPNG | | TPFG | | |
|---|---|---|---|---|---|
| | Relative abundance (%) | | Relative abundance (%) | | Paired t-test |
| Derived Traits | Mean | SEM | Mean | SEM | p-value |
| High mannose glycans | 0.9 | 0.1 | 1.4 | 0.2 | 0.0489 |
| Hybrid glycans | 1.4 | 0.1 | 1.3 | 0.2 | NS |
| Complex glycans | 96.4 | 0.1 | 94.5 | 0.6 | NS |
| Monoantennary glycans | 2.6 | 0.0 | 3.8 | 0.3 | NS |
| Diantennary glycans | 87.3 | 0.3 | 85.6 | 0.7 | NS |
| Triantennary glycans | 9.1 | 0.4 | 8.8 | 0.9 | NS |
| Tetraantennary glycans | 0.1 | 0.0 | 0.2 | 0.1 | NS |
| galactosylation per antenna across all glycan species | 98.6 | 0.1 | 97.8 | 0.3 | NS |
| Fucosylated glycans | 31.1 | 1.9 | 31.3 | 3.0 | NS |
| Di-fucosylated glycans | 0.2 | 0.0 | 0.3 | 0.0 | NS |
| sialic acids (NeuAc & NeuGc) per antenna across all glycan species | 97.1 | 0.7 | 98.3 | 0.6 | NS |
| NeuGc per antenna across all glycan species | 96.5 | 0.7 | 97.8 | 0.6 | NS |
| α-2,3-linked NeuGc per antenna across all glycan species | 17.9 | 0.4 | 18.9 | 0.8 | NS |
| α-2,6-linked NeuGc per antenna across all glycan species | 78.6 | 0.3 | 78.9 | 0.9 | NS |
| NeuAc per antenna across all glycan species | 0.6 | 0.0 | 0.5 | 0.0 | NS |
| α-2,3-linked NeuAc per antenna across all glycan species | 0.4 | 0.0 | 0.4 | 0.0 | NS |
| α-2,6-linked NeuAc per antenna across all glycan species | 0.2 | 0.0 | 0.1 | 0.0 | NS |
| Acetylation of sialic acids (NeuAc & NeuGc) | 7.8 | 0.6 | 10.7 | 0.2 | 0.0158 |
| Branching sialic acids (NeuGc) across all glycan species | 6.1 | 0.3 | 8.0 | 0.1 | 0.0281 |

Supplementary Table S4: Relative abundances of the TPNG and TPFG derived *N* -glycosylation traits in control and zymosan induced-peritonitis (ZIP) mice  (n = 3 per group) at 0h and 24h post-injection. Statictical analysis was performed by one-way ANOVA with a post-hoc Tukey multiple comparison.  NeuAc= *N* -acetylneuraminic acid; NeuGc= *N* -glycolylneuraminic acid; NS= not significant.

## Total Plasma *N*-glycome (TPNG)

| | A- 0 Hour | | B- 24 Hours Control mice | | C- 24 Hours ZIP | | | | | |
|---|---|---|---|---|---|---|---|---|---|---|
| | Relative abundance (%) | | Relative abundance (%) | | Relative abundance (%) | | One-way ANOVA | Multiple comparison | | |
| | | | | | | | | A vs B | A vs C | B vs C |
| Derived Traits | Mean | SEM | Mean | SEM | Mean | SEM | *p*-value | | | |
| High mannose glycans | 0.9 | 0.1 | 0.87 | 0.1 | 1.3 | 0.0 | 0.0213 | NS | 0.0434 | 0.0258 |
| Hybrid glycans | 1.4 | 0.1 | 1.48 | 0.1 | 1.3 | 0.0 | NS | NS | NS | NS |
| Complex glycans | 96.4 | 0.1 | 96.41 | 0.2 | 95.9 | 0.1 | NS | NS | NS | NS |
| Monoantennary glycans | 2.6 | 0.0 | 2.68 | 0.1 | 2.7 | 0.1 | NS | NS | NS | NS |
| Diantennary glycans | 87.3 | 0.3 | 87.57 | 1.0 | 84.1 | 0.4 | 0.0148 | NS | 0.0285 | 0.0192 |
| Triantennary glycans | 9.1 | 0.4 | 8.73 | 0.9 | 11.7 | 0.4 | 0.0237 | NS | 0.0465 | 0.0290 |
| Tetraantennary glycans | 0.1 | 0.0 | 0.11 | 0.0 | 0.1 | 0.0 | NS | NS | NS | NS |
| galactosylation per antenna across all glycan species | 98.6 | 0.1 | 98.63 | 0.2 | 99.0 | 0.1 | NS | NS | NS | NS |
| Fucosylated glycans | 31.1 | 1.9 | 31.95 | 4.1 | 19.7 | 0.5 | 0.0285 | NS | 0.0491 | 0.0376 |
| Di-fucosylated glycans | 0.2 | 0.0 | 0.22 | 0.0 | 0.3 | 0.0 | NS | NS | NS | NS |
| sialic acids (NeuAc & NeuGc) per antenna across all glycan species | 97.1 | 0.7 | 99.05 | 0.3 | 105.6 | 0.3 | < 0,0001 | NS | < 0,0001 | 0.0001 |
| NeuGc per antenna across all glycan species | 96.5 | 0.7 | 98.47 | 0.3 | 104.6 | 0.3 | < 0,0001 | NS | < 0,0001 | 0.0002 |
| $\alpha$-2,3-linked NeuGc per antenna across all glycan species | 17.9 | 0.4 | 17.00 | 1.4 | 24.6 | 0.3 | 0.0012 | NS | 0.0031 | 0.0016 |
| $\alpha$-2,6-linked NeuGc per antenna across all glycan species | 78.6 | 0.3 | 81.47 | 1.1 | 80.0 | 0.2 | NS | NS | NS | NS |

| | | | | | | | | | | |
|---|---|---|---|---|---|---|---|---|---|---|
| NeuAc per antenna across all glycan species | 0.6 | 0.0 | 0.57 | 0.0 | 1.0 | 0.0 | < 0,0001 | NS | 0.0001 | < 0,0001 |
| $\alpha$-2,3--linked NeuAc per antenna across all glycan species | 0.4 | 0.0 | 0.40 | 0.0 | 0.7 | 0.0 | < 0,0001 | NS | < 0,0001 | < 0,0001 |
| $\alpha$-2,6-linked NeuAc per antenna across all glycan species | 0.2 | 0.0 | 0.17 | 0.0 | 0.3 | 0.0 | 0.0040 | NS | 0.0067 | 0.0067 |
| Acetylation of sialic acids (NeuAc & NeuGc) | 7.8 | 0.6 | 8.40 | 0.6 | 6.0 | 0.1 | 0.0328 | NS | NS | 0.0325 |
| Branching sialic acids (NeuGc) across all glycan species | 6.1 | 0.3 | 6.77 | 0.2 | 18.8 | 0.5 | < 0,0001 | NS | < 0,0001 | < 0,0001 |

## Total Peritoneal Fluid *N*-glycome (TPFG)

| | **A- 0 Hour** | | **B- 24 Hours Control mice** | | **C- 24 Hours ZIP** | | | | | |
|---|---|---|---|---|---|---|---|---|---|---|
| | **Relative abundance (%)** | | **Relative abundance (%)** | | **Relative abundance (%)** | | **ANOVA** | **Multiple comparison** | | |
| | | | | | | | | **A vs B** | **A vs C** | **B vs C** |
| **Derived Traits** | **Mean** | **SEM** | **Mean** | **SEM** | **Mean** | **SEM** | ***p*-value** | | | |
| High mannose glycans | 1.4 | 0.2 | 1.60 | 0.1 | 0.7 | 0.2 | 0.0273 | NS | 0.0643 | 0.0296 |
| Hybrid glycans | 1.3 | 0.2 | 1.48 | 0.1 | 1.0 | 0.2 | NS | NS | NS | NS |
| Complex glycans | 94.5 | 0.6 | 94.31 | 0.1 | 96.2 | 1.1 | NS | NS | NS | NS |
| Monoantennary glycans | 3.8 | 0.3 | 3.86 | 0.0 | 3.0 | 0.9 | NS | NS | NS | NS |
| Diantennary glycans | 85.6 | 0.7 | 86.15 | 0.1 | 83.5 | 1.8 | NS | NS | NS | NS |
| Triantennary glycans | 8.8 | 0.9 | 8.04 | 0.1 | 12.4 | 0.7 | 0.0068 | NS | 0.0186 | 0.0076 |
| Tetraantennary glycans | 0.2 | 0.0 | 0.12 | 0.0 | 0.2 | 0.1 | NS | NS | NS | NS |
| galactosylation per antenna across all glycan species | 97.8 | 0.3 | 97.95 | 0.2 | 98.6 | 0.3 | NS | NS | NS | NS |

| | | | | | | | | | | |
|---|---|---|---|---|---|---|---|---|---|---|
| Fucosylated glycans | 31.3 | 3.0 | 30.74 | 4.6 | 22.1 | 1.0 | NS | NS | NS | NS |
| Di-fucosylated glycans | 0.3 | 0.0 | 0.29 | 0.0 | 0.5 | 0.1 | 0.0306 | NS | 0.0415 | NS |
| Bisection | 0.6 | 0.1 | 0.51 | 0.1 | 0.6 | 0.2 | NS | NS | NS | NS |
| sialic acids (NeuAc & NeuGc) per antenna across all glycan species | 98.3 | 0.6 | 98.54 | 0.6 | 105.6 | 0.5 | 0.0002 | NS | 0.0003 | 0.0003 |
| NeuGc per antenna across all glycan species | 97.8 | 0.6 | 97.79 | 0.7 | 104.6 | 0.5 | 0.0003 | NS | 0.0005 | 0.0005 |
| α-2,3-linked NeuGc per antenna across all glycan species | 18.9 | 0.8 | 17.78 | 0.4 | 25.6 | 0.8 | 0.0003 | NS | 0.0008 | 0.0004 |
| α-2,6-linked NeuGc per antenna across all glycan species | 78.9 | 0.9 | 80.01 | 0.7 | 79.0 | 1.1 | NS | NS | NS | NS |
| NeuAc per antenna across all glycan species | 0.5 | 0.0 | 0.75 | 0.0 | 1.0 | 0.0 | 0.0004 | NS | 0.0004 | 0.0061 |
| α-2,3--linked NeuAc per antenna across all glycan species | 0.4 | 0.0 | 0.50 | 0.0 | 0.7 | 0.0 | 0.0002 | NS | 0.0002 | 0.0012 |
| α-2,6-linked NeuAc per antenna across all glycan species | 0.1 | 0.0 | 0.25 | 0.0 | 0.3 | 0.0 | 0.0047 | 0.0318 | 0.0040 | NS |
| Acetylation of sialic acids (NeuAc & NeuGc) | 10.7 | 0.2 | 9.73 | 0.6 | 7.6 | 0.4 | 0.0056 | NS | 0.0051 | 0.0289 |
| Branching sialic acids (NeuGc) across all glycan species | 8.0 | 0.1 | 8.12 | 0.2 | 18.9 | 0.7 | < 0,0001 | NS | < 0,0001 | < 0,0001 |

Supplementary Table S5: Relative abundances of fucosylation, sialylation, α2,3-linked NeuGc and branched sialylation in control and zymosan induced-peritonitis mice (n = 2-3 per group) in the course of zymosan-induced peritonitis . The multiple comparison of glycosylation data between control mice data and zymosan-induced peritonitis mices of experiment 1 (ZIP-exp 1) and experiment 2 (ZIP-exp 2) was made using a two-way analysis of variance (ANOVA) following by a Dunnett test (n=3). NS= not significant. ND= not determined

Fucosylation

| Total Plasma *N* -glycome (TPNG) | | | | | | | | |
|---|---|---|---|---|---|---|---|---|
| | A- Controls | | B- ZIP-exp 1 | | C- ZIP-exp 2 | | | |
| | Relative abundance (%) | | Relative abundance (%) | | Relative abundance (%) | | Two-way ANOVA Multiple comparison | |
| Hours | Mean | SEM | Mean | SEM | Mean | SEM | A vs B | A vs C |
| 0 | 31.1 | 1.9 | 31.3 | 3.0 | 26.4 | 1.8 | NS | NS |
| 2 | 28.8 | 2.8 | 30.7 | 2.5 | 29.0 | 2.6 | NS | NS |
| 4 | 28.5 | 1.6 | 26.8 | 1.7 | 27.4 | 3.0 | NS | NS |
| 12 | 31.6 | 1.1 | 23.5 | 1.5 | 21.5 | 0.7 | 0.0024 | 0.0149 |
| 24 | 32.0 | 4.1 | 19.7 | 0.5 | 19.9 | 1.6 | 0.0003 | 0.0003 |
| 48 | 28.9 | 2.2 | 21.8 | 0.6 | 21.3 | 0.4 | 0.0243 | 0.0345 |
| 72 | 29.7 | 1.6 | 19.4 | 1.7 | 23.1 | 1.8 | 0.002 | NS |

| Total Peritoneal Fluid *N* -glycome (TPFG) | | | | | | | | |
|---|---|---|---|---|---|---|---|---|
| | A- Controls | | B- ZIP-exp 1 | | C- ZIP-exp 2 | | | |
| | Relative abundance (%) | | Relative abundance (%) | | Relative abundance (%) | | Two-way ANOVA Multiple comparison | |
| Hours | Mean | SEM | Mean | SEM | Mean | SEM | A vs B | A vs C |
| 0 | 31.3 | 3.0 | 31.3 | 3.0 | 27.2 | 1.8 | NS | NS |
| 2 | 29.3 | 2.4 | 31.1 | 2.7 | 28.4 | 1.2 | NS | NS |
| 4 | 28.8 | 1.7 | 28.3 | 3.5 | 26.1 | 2.6 | ND | ND |
| 12 | 31.1 | 0.8 | 25.6 | 0.8 | 22.8 | 1.1 | NS | 0.0148 |
| 24 | 30.7 | 4.6 | 22.1 | 1.0 | 19.7 | 1.8 | 0.0117 | 0.0013 |
| 48 | 28.7 | 2.6 | 21.6 | 0.7 | 20.9 | 0.8 | 0.0414 | 0.0235 |
| 72 | 29.2 | 1.1 | 20.3 | 1.8 | 21.6 | 1.6 | 0.0088 | 0.0272 |

Sialylation

## Total Plasma *N*-glycome (TPNG)

| | A- Controls | | B- ZIP-exp 1 | | C- ZIP-exp 2 | | | |
|---|---|---|---|---|---|---|---|---|
| | Relative abundance (%) | | Relative abundance (%) | | Relative abundance (%) | | Two-way ANOVA Multiple comparison | |
| **Hours** | **Mean** | **SEM** | **Mean** | **SEM** | **Mean** | **SEM** | **A vs B** | **A vs C** |
| 0 | 97.1 | 0.7 | 97.1 | 0.7 | 95.1 | 0.4 | NS | NS |
| 2 | 98.5 | 0.5 | 98.5 | 0.8 | 96.5 | 1.3 | NS | NS |
| 4 | 95.1 | 2.9 | 97.8 | 1.0 | 96.8 | 0.6 | NS | NS |
| 12 | 97.7 | 0.4 | 101.6 | 0.4 | 101.2 | 0.5 | 0.0346 | 0.061 |
| 24 | 99.1 | 0.3 | 105.6 | 0.3 | 103.9 | 1.4 | 0.0004 | 0.0087 |
| 48 | 99.0 | 0.2 | 107.0 | 0.8 | 103.6 | 1.4 | < 0.0001 | 0.0134 |
| 72 | 99.0 | 0.4 | 105.2 | 1.5 | 100.7 | 2.5 | 0.0009 | NS |

## Total Peritoneal Fluid *N*-glycome (TPFG)

| | A- Controls | | B- ZIP-exp 1 | | C- ZIP-exp 2 | | | |
|---|---|---|---|---|---|---|---|---|
| | Relative abundance (%) | | Relative abundance (%) | | Relative abundance (%) | | Two-way ANOVA Multiple comparison | |
| **Hours** | **Mean** | **SEM** | **Mean** | **SEM** | **Mean** | **SEM** | **A vs B** | **A vs C** |
| 0 | 98.3 | 0.6 | 98.3 | 0.6 | 98.8 | 0.7 | NS | NS |
| 2 | 98.4 | 0.5 | 99.2 | 0.4 | 100.0 | 0.1 | NS | NS |
| 4 | 98.7 | 0.2 | 100.4 | 1.4 | 99.8 | 0.2 | ND | ND |
| 12 | 98.9 | 0.5 | 101.5 | 0.5 | 102.8 | 0.2 | 0.0055 | < 0.0001 |
| 24 | 98.5 | 0.6 | 105.6 | 0.5 | 105.6 | 0.4 | < 0.0001 | < 0.0001 |
| 48 | 99.8 | 0.2 | 108.9 | 0.5 | 107.6 | 0.7 | < 0.0001 | < 0.0001 |
| 72 | 99.3 | 0.9 | 106.7 | 0.2 | 105.1 | 1.0 | < 0.0001 | < 0.0001 |

## α-2,3-linked NeuGc

### Total Plasma *N*-glycome (TPNG)

| | A- Controls | | B- ZIP-exp 1 | | C- ZIP-exp 2 | | | |
|---|---|---|---|---|---|---|---|---|
| | Relative abundance (%) | | Relative abundance (%) | | Relative abundance (%) | | Two-way ANOVA Multiple comparison | |
| Hours | Mean | SEM | Mean | SEM | Mean | SEM | A vs B | A vs C |
| 0 | 17.9 | 0.4 | 17.9 | 0.4 | 18.3 | 0.8 | NS | NS |
| 2 | 18.8 | 0.3 | 19.0 | 0.8 | 19.0 | 0.8 | NS | NS |
| 4 | 17.5 | 1.4 | 19.8 | 0.7 | 17.8 | 0.4 | NS | NS |
| 12 | 16.3 | 0.3 | 20.9 | 0.4 | 21.6 | 0.0 | 0.0035 | 0.0008 |
| 24 | 17.0 | 1.4 | 24.6 | 0.3 | 24.2 | 0.9 | < 0.0001 | < 0.0001 |
| 48 | 18.4 | 0.5 | 25.5 | 0.8 | 24.7 | 1.6 | < 0.0001 | < 0.0001 |
| 72 | 19.0 | 0.4 | 25.9 | 2.2 | 22.3 | 1.8 | < 0.0001 | 0.0378 |

### Total Peritoneal Fluid *N*-glycome (TPFG)

| | A- Controls | | B- ZIP-exp 1 | | C- ZIP-exp 2 | | | |
|---|---|---|---|---|---|---|---|---|
| | Relative abundance (%) | | Relative abundance (%) | | Relative abundance (%) | | Two-way ANOVA Multiple comparison | |
| Hours | Mean | SEM | Mean | SEM | Mean | SEM | A vs B | A vs C |
| 0 | 18.9 | 0.8 | 18.9 | 0.8 | 20.4 | 1.4 | NS | NS |
| 2 | 18.3 | 1.0 | 18.4 | 1.1 | 20.0 | 0.5 | NS | NS |
| 4 | 19.9 | 0.6 | 20.2 | 0.0 | 19.7 | 0.5 | ND | ND |
| 12 | 17.9 | 0.8 | 19.9 | 1.0 | 22.6 | 0.4 | 0.1818 | 0.0008 |
| 24 | 17.8 | 0.4 | 25.6 | 0.8 | 25.5 | 0.4 | < 0.0001 | < 0.0001 |
| 48 | 19.6 | 1.0 | 27.5 | 0.7 | 26.8 | 0.6 | < 0.0001 | < 0.0001 |
| 72 | 21.1 | 0.8 | 25.4 | 1.0 | 25.7 | 1.2 | 0.0023 | 0.0011 |

Branching sialic acids

**Total Plasma *N* -glycome (TPNG)**

| | A- Controls | | B- ZIP-exp 1 | | C- ZIP-exp 2 | | Two-way ANOVA Multiple comparison | |
|---|---|---|---|---|---|---|---|---|
| | Relative abundance (%) | | Relative abundance (%) | | Relative abundance (%) | | | |
| Hours | Mean | SEM | Mean | SEM | Mean | SEM | A vs B | A vs C |
| 0 | 6.1 | 0.3 | 6.1 | 0.3 | 6.9 | 0.4 | NS | NS |
| 2 | 6.6 | 0.1 | 6.6 | 0.2 | 6.9 | 0.4 | NS | NS |
| 4 | 5.9 | 0.6 | 7.8 | 0.7 | 7.3 | 0.3 | NS | NS |
| 12 | 6.5 | 0.2 | 12.4 | 0.6 | 14.5 | 0.6 | < 0.0001 | < 0.0001 |
| 24 | 6.8 | 0.2 | 18.8 | 0.5 | 17.2 | 0.2 | < 0.0001 | < 0.0001 |
| 48 | 6.8 | 0.1 | 21.0 | 0.9 | 17.6 | 1.8 | < 0.0001 | < 0.0001 |
| 72 | 6.7 | 0.4 | 17.9 | 1.5 | 14.8 | 1.7 | < 0.0001 | < 0.0001 |

**Total Peritoneal Fluid *N* -glycome (TPFG)**

| | A- Controls | | B- ZIP-exp 1 | | C- ZIP-exp 2 | | Two-way ANOVA Multiple comparison | |
|---|---|---|---|---|---|---|---|---|
| | Relative abundance (%) | | Relative abundance (%) | | Relative abundance (%) | | | |
| Hours | Mean | SEM | Mean | SEM | Mean | SEM | A vs B | A vs C |
| 0 | 8.0 | 0.1 | 8.0 | 0.1 | 8.0 | 0.5 | NS | NS |
| 2 | 6.7 | 0.3 | 7.0 | 0.6 | 7.3 | 0.1 | NS | NS |
| 4 | 7.4 | 0.5 | 8.5 | 1.3 | 7.3 | 0.1 | ND | ND |
| 12 | 8.1 | 0.3 | 11.2 | 0.6 | 12.3 | 0.3 | 0.002 | < 0.0001 |
| 24 | 8.1 | 0.2 | 18.9 | 0.7 | 17.5 | 0.6 | < 0.0001 | < 0.0001 |
| 48 | 8.0 | 0.3 | 23.7 | 0.7 | 20.5 | 0.9 | < 0.0001 | < 0.0001 |
| 72 | 9.7 | 1.1 | 18.9 | 0.4 | 16.3 | 1.3 | < 0.0001 | < 0.0001 |

Supplementary Table S6: Relative abundances of fucosylation, galactosylation and sialylation levels in IgG subclasses (IgG1, IgG2 and IgG3) isolated from the plasma and peritoneal fluid of mice in response to zymosan-induced peritonitis (ZIP) or PBS injection (controls).

IgG1 Fucosylation

| Total Plasma *N*-glycome (TPNG) | | | | |
|---|---|---|---|---|
| | A- Controls | | B- ZIP-exp 1 | |
| | Relative abundance (%) | | Relative abundance (%) | |
| Hours | Mean | SEM | Mean | SEM |
| 0 | 99.7 | 0.1 | 99.7 | 0.1 |
| 2 | 99.8 | 0.0 | 99.8 | 0.0 |
| 4 | 99.6 | 0.2 | 99.8 | 0.0 |
| 12 | 99.8 | 0.0 | 99.7 | 0.1 |
| 24 | 99.7 | 0.1 | 99.8 | 0.1 |
| 48 | 99.8 | 0.0 | 99.7 | 0.0 |
| 72 | 99.7 | 0.0 | 99.7 | 0.0 |
| **Total Peritoneal Fluid *N*-glycome (TPFG)** | | | | |
| | A- Controls | | B- ZIP-exp 1 | |
| | Relative abundance (%) | | Relative abundance (%) | |
| Hours | Mean | SEM | Mean | SEM |
| 0 | 99.8 | 0.0 | 99.8 | 0.0 |
| 2 | 99.9 | 0.0 | 99.9 | 0.0 |
| 4 | 99.9 | 0.0 | 99.9 | 0.0 |
| 12 | 99.9 | 0.0 | 99.8 | 0.0 |
| 24 | 99.8 | 0.0 | 99.8 | 0.0 |
| 48 | 99.8 | 0.0 | 99.8 | 0.0 |
| 72 | 99.9 | 0.0 | 99.8 | 0.0 |

IgG1 Galactosylation

| Total Plasma *N*-glycome (TPNG) | | | | |
|---|---|---|---|---|
| | A- Controls | | B- ZIP-exp 1 | |
| | Relative abundance (%) | | Relative abundance (%) | |
| Hours | Mean | SEM | Mean | SEM |
| 0 | 25.5 | 1.3 | 25.5 | 1.3 |
| 2 | 25.4 | 0.5 | 26.5 | 0.7 |
| 4 | 27.1 | 0.6 | 27.9 | 1.4 |
| 12 | 24.3 | 1.8 | 25.8 | 1.3 |
| 24 | 27.2 | 1.7 | 25.4 | 0.8 |
| 48 | 26.7 | 1.1 | 29.3 | 1.1 |
| 72 | 25.3 | 1.1 | 26.6 | 2.5 |

| Total Peritoneal Fluid *N*-glycome (TPFG) | | | | |
|---|---|---|---|---|
| | A- Controls | | B- ZIP-exp 1 | |
| | Relative abundance (%) | | Relative abundance (%) | |
| Hours | Mean | SEM | Mean | SEM |
| 0 | 25.4 | 1.2 | 25.4 | 1.2 |
| 2 | 24.6 | 0.5 | 25.3 | 0.5 |
| 4 | 26.4 | 0.8 | 27.0 | 1.4 |
| 12 | 24.4 | 1.4 | 23.9 | 1.1 |
| 24 | 26.7 | 1.6 | 24.6 | 0.9 |
| 48 | 26.0 | 1.1 | 28.4 | 1.2 |
| 72 | 24.9 | 0.9 | 25.8 | 2.3 |

IgG1 Sialylation

| Total Plasma *N*-glycome (TPNG) | | | | |
|---|---|---|---|---|
| | **A- Controls** | | **B- ZIP-exp 1** | |
| | **Relative abundance (%)** | | **Relative abundance (%)** | |
| **Hours** | **Mean** | **SEM** | **Mean** | **SEM** |
| 0 | 8.8 | 0.6 | 8.8 | 0.6 |
| 2 | 8.5 | 0.4 | 9.0 | 0.2 |
| 4 | 9.2 | 0.4 | 8.6 | 0.3 |
| 12 | 8.4 | 0.6 | 8.7 | 0.2 |
| 24 | 9.3 | 0.5 | 8.7 | 0.6 |
| 48 | 9.0 | 0.5 | 10.1 | 0.4 |
| 72 | 8.5 | 0.4 | 9.2 | 1.0 |

| Total Peritoneal Fluid *N*-glycome (TPFG) | | | | |
|---|---|---|---|---|
| | **A- Controls** | | **B- ZIP-exp 1** | |
| | **Relative abundance (%)** | | **Relative abundance (%)** | |
| **Hours** | **Mean** | **SEM** | **Mean** | **SEM** |
| 0 | 8.6 | 0.5 | 8.6 | 0.5 |
| 2 | 8.3 | 0.5 | 9.2 | 0.3 |
| 4 | 9.1 | 0.5 | 8.6 | 0.5 |
| 12 | 8.3 | 0.4 | 8.1 | 0.1 |
| 24 | 9.1 | 0.5 | 8.4 | 0.5 |
| 48 | 8.6 | 0.6 | 9.9 | 0.4 |
| 72 | 8.4 | 0.4 | 8.9 | 0.9 |

IgG2 Fucosylation

| Total Plasma *N* -glycome (TPNG) | | | | |
|---|---|---|---|---|
| | A- Controls | | B- ZIP-exp 1 | |
| | Relative abundance (%) | | Relative abundance (%) | |
| Hours | Mean | SEM | Mean | SEM |
| 0 | 99.2 | 0.6 | 99.2 | 0.6 |
| 2 | 99.8 | 0.1 | 99.7 | 0.0 |
| 4 | 99.7 | 0.0 | 99.8 | 0.0 |
| 12 | 99.7 | 0.0 | 99.8 | 0.1 |
| 24 | 99.9 | 0.0 | 99.7 | 0.0 |
| 48 | 99.7 | 0.1 | 99.8 | 0.0 |
| 72 | 99.8 | 0.0 | 99.8 | 0.0 |
| **Total Peritoneal Fluid *N*-glycome (TPFG)** | | | | |
| | A- Controls | | B- ZIP-exp 1 | |
| | Relative abundance (%) | | Relative abundance (%) | |
| Hours | Mean | SEM | Mean | SEM |
| 0 | 99.7 | 0.1 | 99.7 | 0.1 |
| 2 | 99.7 | 0.1 | 99.7 | 0.0 |
| 4 | 99.8 | 0.0 | 99.8 | 0.0 |
| 12 | 99.8 | 0.0 | 99.6 | 0.1 |
| 24 | 99.8 | 0.0 | 99.7 | 0.1 |
| 48 | 99.8 | 0.0 | 99.8 | 0.0 |
| 72 | 99.8 | 0.0 | 99.7 | 0.0 |

IgG2 Galactosylation

| Total Plasma *N*-glycome (TPNG) | | | | |
|---|---|---|---|---|
| | A- Controls | | B- ZIP-exp 1 | |
| | Relative abundance (%) | | Relative abundance (%) | |
| Hours | Mean | SEM | Mean | SEM |
| 0 | 64.2 | 1.2 | 64.2 | 1.2 |
| 2 | 61.0 | 1.5 | 61.9 | 0.8 |
| 4 | 62.3 | 3.0 | 63.2 | 1.5 |
| 12 | 63.5 | 1.8 | 64.4 | 1.0 |
| 24 | 62.0 | 0.5 | 61.4 | 0.9 |
| 48 | 63.2 | 1.2 | 63.3 | 1.4 |
| 72 | 64.4 | 0.9 | 63.1 | 2.4 |
| **Total Peritoneal Fluid *N*-glycome (TPFG)** | | | | |
| | A- Controls | | B- ZIP-exp 1 | |
| | Relative abundance (%) | | Relative abundance (%) | |
| Hours | Mean | SEM | Mean | SEM |
| 0 | 63.5 | 1.3 | 63.5 | 1.3 |
| 2 | 60.8 | 1.5 | 62.1 | 0.8 |
| 4 | 62.1 | 3.3 | 62.1 | 2.3 |
| 12 | 63.5 | 1.9 | 64.0 | 1.6 |
| 24 | 62.8 | 0.5 | 61.2 | 0.9 |
| 48 | 62.7 | 1.1 | 62.9 | 1.3 |
| 72 | 64.2 | 0.5 | 62.5 | 2.3 |

IgG2 Sialylation

## Total Plasma *N*-glycome (TPNG)

| | A- Controls | | B- ZIP-exp 1 | |
|---|---|---|---|---|
| | Relative abundance (%) | | Relative abundance (%) | |
| Hours | Mean | SEM | Mean | SEM |
| 0 | 22.4 | 0.6 | 22.4 | 0.6 |
| 2 | 21.3 | 0.9 | 21.6 | 0.4 |
| 4 | 21.3 | 1.1 | 21.6 | 0.7 |
| 12 | 21.8 | 1.1 | 22.1 | 0.2 |
| 24 | 19.5 | 1.4 | 21.0 | 0.3 |
| 48 | 22.1 | 0.7 | 21.3 | 0.7 |
| 72 | 23.0 | 0.7 | 20.9 | 0.9 |

## Total Peritoneal Fluid *N*-glycome (TPFG)

| | A- Controls | | B- ZIP-exp 1 | |
|---|---|---|---|---|
| | Relative abundance (%) | | Relative abundance (%) | |
| Hours | Mean | SEM | Mean | SEM |
| 0 | 22.1 | 0.8 | 22.1 | 0.8 |
| 2 | 21.7 | 1.1 | 23.3 | 0.2 |
| 4 | 21.3 | 1.6 | 22.1 | 1.3 |
| 12 | 21.8 | 0.8 | 23.0 | 0.9 |
| 24 | 21.9 | 0.2 | 20.9 | 0.5 |
| 48 | 22.0 | 0.9 | 21.7 | 0.8 |
| 72 | 22.6 | 0.4 | 21.8 | 1.0 |

IgG3 Fucosylation

| Total Plasma *N*-glycome (TPNG) | | | | |
|---|---|---|---|---|
| | A- Controls | | B- ZIP-exp 1 | |
| | Relative abundance (%) | | Relative abundance (%) | |
| Hours | Mean | SEM | Mean | SEM |
| 0 | 99.7 | 0.1 | 99.7 | 0.1 |
| 2 | 99.7 | 0.0 | 99.5 | 0.1 |
| 4 | 99.7 | 0.1 | 99.8 | 0.1 |
| 12 | 99.8 | 0.0 | 99.2 | 0.2 |
| 24 | 99.4 | 0.2 | 99.3 | 0.2 |
| 48 | 99.6 | 0.1 | 99.4 | 0.5 |
| 72 | 99.7 | 0.1 | 99.7 | 0.0 |

| Total Peritoneal Fluid *N*-glycome (TPFG) | | | | |
|---|---|---|---|---|
| | A- Controls | | B- ZIP-exp 1 | |
| | Relative abundance (%) | | Relative abundance (%) | |
| Hours | Mean | SEM | Mean | SEM |
| 0 | 99.8 | 0.0 | 99.8 | 0.0 |
| 2 | 99.8 | 0.0 | 99.9 | 0.0 |
| 4 | 99.8 | 0.0 | 99.9 | 0.0 |
| 12 | 99.8 | 0.0 | 99.8 | 0.0 |
| 24 | 99.8 | 0.0 | 99.8 | 0.1 |
| 48 | 99.8 | 0.0 | 99.7 | 0.0 |
| 72 | 99.7 | 0.1 | 99.7 | 0.0 |

IgG3 Galactosylation

| Total Plasma *N*-glycome (TPNG) | | | | |
|---|---|---|---|---|
| | A- Controls | | B- ZIP-exp 1 | |
| | Relative abundance (%) | | Relative abundance (%) | |
| Hours | Mean | SEM | Mean | SEM |
| 0 | 45.7 | 1.7 | 45.7 | 1.7 |
| 2 | 48.3 | 0.9 | 51.6 | 0.5 |
| 4 | 47.2 | 1.8 | 49.5 | 0.5 |
| 12 | 48.1 | 1.1 | 42.1 | 3.9 |
| 24 | 48.3 | 3.1 | 51.2 | 1.7 |
| 48 | 48.8 | 1.9 | 45.8 | 3.1 |
| 72 | 43.8 | 0.3 | 44.7 | 1.2 |
| **Total Peritoneal Fluid *N*-glycome (TPFG)** | | | | |
| | A- Controls | | B- ZIP-exp 1 | |
| | Relative abundance (%) | | Relative abundance (%) | |
| Hours | Mean | SEM | Mean | SEM |
| 0 | 44.9 | 1.8 | 44.9 | 1.8 |
| 2 | 48.1 | 1.0 | 50.1 | 0.8 |
| 4 | 47.3 | 1.5 | 48.3 | 0.2 |
| 12 | 47.6 | 0.9 | 41.1 | 4.2 |
| 24 | 46.9 | 2.9 | 50.4 | 1.9 |
| 48 | 47.9 | 2.2 | 44.6 | 3.1 |
| 72 | 43.5 | 0.5 | 44.2 | 0.9 |

IgG3 Sialylation

| Total Plasma *N* -glycome (TPNG) | | | | |
|---|---|---|---|---|
| | A- Controls | | B- ZIP-exp 1 | |
| | Relative abundance (%) | | Relative abundance (%) | |
| **Hours** | **Mean** | **SEM** | **Mean** | **SEM** |
| 0 | 13.7 | 1.1 | 13.7 | 1.1 |
| 2 | 15.0 | 0.4 | 16.9 | 0.1 |
| 4 | 13.3 | 1.5 | 14.6 | 0.2 |
| 12 | 14.0 | 0.9 | 11.8 | 1.7 |
| 24 | 15.3 | 1.9 | 16.3 | 0.9 |
| 48 | 14.8 | 1.2 | 13.3 | 1.4 |
| 72 | 12.8 | 0.2 | 11.9 | 0.5 |

| Total Peritoneal Fluid *N*-glycome (TPFG) | | | | |
|---|---|---|---|---|
| | A- Controls | | B- ZIP-exp 1 | |
| | Relative abundance (%) | | Relative abundance (%) | |
| **Hours** | **Mean** | **SEM** | **Mean** | **SEM** |
| 0 | 13.6 | 1.0 | 13.6 | 1.0 |
| 2 | 15.1 | 0.6 | 15.9 | 0.1 |
| 4 | 13.7 | 1.3 | 14.3 | 0.1 |
| 12 | 13.9 | 0.8 | 11.2 | 1.9 |
| 24 | 14.3 | 1.8 | 15.7 | 0.9 |
| 48 | 14.5 | 1.3 | 12.8 | 1.4 |
| 72 | 12.5 | 0.4 | 11.8 | 0.5 |
